# A circuit from lateral septum neurotensin neurons to tuberal nucleus controls hedonic feeding

**DOI:** 10.1038/s41380-022-01742-0

**Published:** 2022-08-26

**Authors:** Zijun Chen, Gaowei Chen, Jiafeng Zhong, Shaolei Jiang, Shishi Lai, Hua Xu, Xiaofei Deng, Fengling Li, Shanshan Lu, Kuikui Zhou, Changlin Li, Zhongdong Liu, Xu Zhang, Yingjie Zhu

**Affiliations:** 1 Shenzhen Key Laboratory of Drug Addiction, Shenzhen Neher Neural Plasticity Laboratory, the Brain Cognition and Brain Disease Institute, Shenzhen Institute of Advanced Technology, Chinese Academy of Sciences; Shenzhen-Hong Kong Institute of Brain Science-Shenzhen Fundamental Research Institutions, Shenzhen, 518055 China; 2 University of Chinese Academy of Sciences, 100049 Beijing, China; 3 Henan University of Technology, Henan, 450001 China; 4 University of Shanghai for Science and Technology, Shanghai, 200093 China; 5 Faculty of Life and Health Sciences, Shenzhen Institute of Advanced Technology, Chinese Academy of Sciences, Shenzhen, 518055 China; 6 Guangdong Institute of Intelligence Science and Technology, Hengqin District, Zhuhai, Guangdong 519031 China; 7 Research Unit of Pain Medicine, Chinese Academy of Medical Sciences; SIMR Joint Lab of Drug Innovation, Shanghai Advanced Research Institute, Chinese Academy of Sciences, Shanghai, 201210 China; 8 CAS Center for Excellence in Brain Science and Intelligence Technology, Chinese Academy of Sciences, Shanghai, 200031 China; 9 CAS Key Laboratory of Brain Connectome and Manipulation, the Brain Cognition and Brain Disease Institute (BCBDI), Shenzhen Institute of Advanced Technology (SIAT), Chinese Academy of Sciences, Shenzhen, 518055 China

**Keywords:** Neuroscience, Physiology

## Abstract

Feeding behavior is regulated by both the homeostatic needs of the body and hedonic values of the food. Easy access to palatable energy-dense foods and the consequent obesity epidemic stress the urgent need for a better understanding of neural circuits that regulate hedonic feeding. Here, we report that neurotensin-positive neurons in the lateral septum (LS^Nts^) play a crucial role in regulating hedonic feeding. Silencing LS^Nts^ specifically promotes feeding of palatable food, whereas activation of LS^Nts^ suppresses overall feeding. LS^Nts^ neurons project to the tuberal nucleus (TU) via GABA signaling to regulate hedonic feeding, while the neurotensin signal from LS^Nts^→the supramammillary nucleus (SUM) is sufficient to suppress overall feeding. In vivo calcium imaging and optogenetic manipulation reveal two populations of LS^Nts^ neurons that are activated and inhibited during feeding, which contribute to food seeking and consumption, respectively. Chronic activation of LS^Nts^ or LS^Nts^→TU is sufficient to reduce high-fat diet-induced obesity. Our findings suggest that LS^Nts^→TU is a key pathway in regulating hedonic feeding.

## Introduction

The incidence of obesity and related metabolic diseases has increased rapidly over the past several decades and has become a major health concern worldwide [1]. A main driving factor underlying the obesity pandemic is overeating caused by the overwhelming availability of highly palatable calorie-dense food in modern society. Feeding can be driven by energy demands, which is an evolutionarily conserved mechanism to maintain metabolic homeostasis. This homeostatic feeding is tightly controlled by the activity of brain networks and circulating hormones [2–4]. On the other hand, hedonic feeding is driven by the pleasure of consuming palatable food without a metabolic need, which is a major factor contributing to overeating and obesity [5].

Although the neural circuits that mediate homeostatic feeding have been extensively studied, much less is known about the neural substrates regulating hedonic feeding [6–8]. Homeostatic and hedonic feeding could be processed by separate and distinct neural circuits [6]. The hypothalamic nuclei, including the arcuate nucleus (ARC) and lateral hypothalamic area (LHA), are well recognized in mediating homeostatic feeding that transforms hunger signals to food seeking and consumption [9]. Generally, hedonic feeding is presumed to be mediated by the mesolimbic dopaminergic reward system, including the ventral tegmental area (VTA) and its target, the nucleus accumbens (NAc) [2, 10, 11]. However, genetically engineered dopamine-deficient mice stop feeding and die within a few weeks after birth [12], suggesting VTA dopamine system also plays crucial roles in regulating behaviors that are important for animals’ survival such as homeostatic feeding. Furthermore, agouti-related peptide (AGRP)-expressing neurons in the ARC are well characterized in controlling homeostatic feeding [13]. Ablation of AGRP neurons abolishes the consumption of regular chow food but had no effect on palatable food intake induced by ghrelin [14]. According to a recent study, activating anterior paraventricular thalamus (aPVT) input to the NAc promotes hedonic feeding of high-fat food but has no effect on overnight chow intake [15]. These studies suggested that distinct neural circuits might differentially contribute to homeostatic and hedonic feeding.

The lateral septum (LS) receives hippocampal inputs and sends massive projections to the hypothalamus; thus, it is particularly well situated for integrating contextual information, such as food palatability, to guide feeding behavior. Previous studies suggested potential roles for the LS in regulating both general feeding and stress-induced anxiety [4, 16]. However, little is known about how LS cell types and circuits contribute to hedonic feeding.

We examined the involvement of the LS in the regulation of hedonic feeding by examining brain activation during the consumption of palatable food without an energy deficit and then compared LS activation during the consumption of regular chow vs. palatable food. Using this approach, we identified a subset of neurotensin-expressing GABAergic neurons in the lateral septum (LS^Nts^) that was specifically activated during hedonic feeding. Silencing LS^Nts^ with tetanus neurotoxin-mediated synaptic inactivation or optogenetic approaches specifically promotes feeding with palatable food. However, activation of LS^Nts^ suppresses overall feeding. Both the canonical neurotransmitter GABA and neuropeptide neurotensin contribute to the modulatory effects of LS^Nts^ neurons on feeding. LS^Nts^ project to the tuberal nucleus (TU) via GABA to regulate hedonic feeding, while the neurotensin signal in LS^Nts^ → the supramammillary nucleus (SUM) is sufficient to suppress overall feeding. The identification of precise molecular, cell type and circuitry may help to develop novel therapeutics targeting hedonic feeding for the treatment of obesity and related metabolic diseases.

## Materials and methods

### Animals

All husbandry and experimental procedures in this study were approved by the Animal Care and Use Committees at the Shenzhen Institute of Advanced Technology (SIAT), Chinese Academy of Sciences (CAS). Adult (3–5-month-old) male C57BL/6 J (Guangdong Medical Laboratory Animal Center, Guangzhou, China), *Nts-ires-Cre* (Jax No. 017525), *Rosa26-LSL-Cas9* (Jax No. 024857) mice were used in this study. Mice were housed at 22–25 °C on a circadian of 12-hour light and 12-hour dark cycle.

### Virus and reagents

We purchased AAV2/9-hSyn-mCherry-P2A-TetTox-WPRE-pA, AAV2/9-hEF1a-DIO--hM3D(Gq)-mCherry-WPRE-pA, AAV2/9-hSyn-DIO-hChR2(H134R)-mCherry-WPRE-pA, AAV2/9-hEF1a-DIO-eNpHR3.0-EYFP, AAV2/9-hSyn-FLEX-GCamP6s-WPRE-pA, AAV2/2Retro-hSyn-DIO-GCaMP6s-WPRE-pA, AAV2/9-hEF1a-DIO-EYFP-WPRE-pA, AAV2/9-hEF1a-DIO-mCherry-WPRE-pA, AAV2/9-hSyn-FLEX-tdTomato-T2A--synaptophysin-EGFP-WPRE-pA, scAAV2/2Retro-hsyn-FLEX-Flpo-pA, AAV2/9-hEF1a--fDIO-hM3D(Gq)-mCherry-WPRE-pA from Tailtool. AAV-U6-sgRNA(LacZ)-pCbh-DIO--hM3D(Gq)-mCherry, AAV-U6-sgRNA(vGAT)-pCbh-DIO-hM3D(Gq)-mCherry and AAV--U6-sgRNA(Nts)-CAG-DIO-hM3D(Gq)-mCherry were made by BrainCase using constructs provided by Y. Dan at University of California Berkeley. The clozapine N-oxide (BML-NS105-0025) was purchased from Enzo. The CTB-488 (C34775), CTB-555 (C34776) and CTB-647 (C34778) were purchased from Thermo Fisher. The c-fos antibody (2250) was purchased from Cell Signaling Technology. The GFP (ab13970) and mCherry (ab167453, ab205402) antibody were purchased from Abcam. The RNA probes and RNAScope in situ hybridization reagent kits were purchased from ACDbio.

### Stereotaxic surgeries

#### Injection of AAV and CTB

Mice were anesthetized with intraperitoneal injection of pentobarbital (80 mg/kg). Standard surgery was performed to expose the brain surface above the LS, POA, AHN, TU or SUM. Coordinates used for LS injection were: bregma +0.75 mm, lateral ±0.35 mm, and dura −2.85 mm. Coordinates used for POA injection were: bregma +0.62 mm, lateral ±0.25 mm, and dura −4.25 mm. Coordinates used for AHN injection were: bregma −0.80 mm, lateral ±0.40 mm, and dura −5.0 mm. Coordinates used for TU injection were: bregma −1.94 mm, lateral ±1.20 mm, and dura −5.10 mm. Coordinates used for SUM injection were: bregma −3.0 mm, lateral ±0.25 mm, and dura −4.4 mm. The AAV vectors and CTB-488/594/633 were stereotaxically injected with a glass pipette connected to Nano-liter Injector (Drummond Scientific Company) at a slow flow rate of 60 nl/min to avoid potential damage of local brain tissue. The pipette was withdrawn at least 10 min after viral injection.

For synaptic inactivation and chemogenetic activation experiments, all injections (AAV-DIO-EGFP-2A-TeNT, AAV-DIO-EYFP, AAV-DIO-hM3D-mCherry, AAV-DIO-mCherry, AAV-Retro-Flex-FlpO, AAV-fDIO-hM3D-mCherry, AAV-sgRNA(LacZ/ vGAT / Nts)-DIO-hM3D-mCherry) were bilateral. Behavioral tests were conducted at 3 weeks after viral injection. For pathway tracing experiments, the AAV (AAV-DIO- tdTomato-T2A-Synaptophysin-EGFP) and CTB injections were unilateral on the same side. Histological analyses were conducted 1 week (for CTB) or 3 weeks (for AAV) after injection. For optogenetic manipulation, fiber photometry and calcium imaging experiments, the AAV injections (AAV-DIO-ChR2-mCherry, AAV-DIO-mCherry, AAV-DIO-eNpHR-EYFP, AAV-DIO-EYFP, AAV-DIO-GCaMP6m) were unilateral and followed by optic fiber or miniature microscope implantation, as described below.

#### Optic fiber and miniature microscope implantation

Thirty minutes after AAV injections, a ceramic ferrule with an optic fiber (200 µm in diameter, N.A. 0.37) was implanted with the fiber tip on top of the LS (bregma +0.75 mm, lateral +0.35 mm, and dura −2.70 mm), TU (bregma −1.94 mm, lateral +1.20 mm, and dura −4.80 mm) or SUM (bregma −3.0 mm, lateral ±0.25 mm, and dura −4.1 mm). The ferrule was then secured on the skull with light-curable resins. After implantation, the skin was sutured and antibiotics were applied to the surgical wound. The optogenetic and fiber photometry experiments were conducted at least 3 weeks after optic fiber implantation.

Miniature microscopy surgery procedure was as described previously (Resendez et al., 2016). Three weeks after AAV-DIO-GCaMP6 AAV injection, GRIN lens (Go!Foton, Cat# CLHS050GFT009) was implanted with the lens tip on top of the LS (bregma +0.75 mm, lateral +0.35 mm, and dura −2.70 mm). The lens was then secured on the skull with light-curable resins. Baseplate was attached 3 weeks after the implantation of GRIN lens. Behavior experiments were started at least one week after recovery from baseplate attachment.

### Behavioral tests

After virus injection or fiber implantation, the mice were housed in groups (3~5 animals per cage) for at least 3 weeks before the behavioral tests. They were handled daily by the experimenters for at least 3 days before the behavioral tests. All behaviors were scored by the experimenters, who were blind to the treatment of the animals. All behavioral experiments were repeated at least 3 times in the laboratory.

#### Solid food intake assays

The mice were housed individually at least 3 days before the solid food intake assays. During feeding behavior experiments, the different kinds of food (standard chow, high-sucrose food or high-fat food, ~20 g) were replaced daily, and cages were changed daily to prevent food debris from gathering at the bottom. Food intake was calculated manually in the home cage during the early dark phase (8:00–10:00 P.M.) by briefly removing the food from the cages and measuring its weight. And the caloric intake was calculated according to burden sheet. To avoid potential effects of stress caused by changing diet, mice were habituated to new diet for at least 3 days.

For c-fos mapping experiment, the feeding experiment were performed with or without energy deficit. For control feeding group, mice were fed with the standard laboratory chow *ad libitum*. For the hedonic feeding group, mice had unlimited access to standard chow while high-sucrose food was provided for 2 h each day for 7 continuous days. Food intake during this 2-hours period was monitored. For feeding induced by acute energy deficit, food was removed for 22 hours (fasted group) and then provided with standard chow (fasted + chow group) or high-sucrose food (fasted + HSF group). The experiment was repeated 3 times in the laboratory.

For TeNT-induced synaptic inactivation and CRISPR/Cas9-mediated knockdown experiments, the intakes for different kinds of food were measured successively in the sated or fasted state, as described above.

For chemogenetic activation experiments, food intake was measured 30 min after intraperitoneal injections of saline (0.9%; 200 μl) or CNO (Catalog No. BML-NS105-0025, Enzo, 2 mg/kg body weight in saline). The intakes for different kinds of food were measured successively in the sated or fasted state.

#### Liquid food intake assays

For the free-liquid food intake assays, mice were individually caged with *ad libitum* food and water before the experiment. During the experiment, the mice were placed in the operant conditioning chamber (22 cm × 16 cm × 15 cm, AniLab). A drop (10 μl) of liquid (water, 10% sucrose solution, Ensure, or starch solution) was delivered every 10 s, with the sequence repeated 180 times. Liquid was pushed out by a pump that held the liquid-containing syringe. Licks were monitored by a custom-made lickometer with a capacitive touch sensor (Sparkfun MPR121) and a microcontroller (Arduino). From day 1 to day 3, mice were trained to lick the spout. For TeNT-induced synaptic inactivation experiments, after 3 days training, the consumption of the different liquid was measured on day 4. For chemogenetic activation experiments, mice were divided into two groups randomly on day 4 and were treated with saline or CNO (2 mg/kg body weight in saline, IP) 30 min before testing. On the next day (day 5), mice were treated with the CNO or saline conversely and tested again. For optogenetic inhibition experiments, a 593-nm diode pumped solid state laser system was used to generate the 593-nm blue laser for light stimulation. An FC/PC adaptor was used to connect the output of the laser to the implanted ferrule for intracranial light delivery. On day 4, mice were placed in the behavior chamber and consumption of liquid food was measured while light was off. On day 5, mice were placed in the same chamber for food intake assay while yellow light stimulation was delivered (593 nm) constantly during the whole feeding session.

For cue-conditioned feeding of liquids (Ensure, regular liquid food, water), a 1-s auditory cue (20 kHz) was presented every 20–24 s after onset of the trial. Immediately after cue presentation, a drop (10 μl) of liquid food was pushed out by a pump that held the liquid-containing syringe. Licks were monitored by a custom-made lickometer with a capacitive touch sensor (Sparkfun MPR121) and a microcontroller (Arduino).

For self-paced free feeding of liquid food, mice had free access to Ensure via the lickometer spout for 30 min in each self-paced feeding session. A drop (10 μl) of Ensure was delivered once a lick was detected. Each self-paced consumption session usually contained multiple consumption bouts. Each bout was defined as continuous licking with an interlick interval less than 6 s. The neuronal dynamics were aligned to the first lick of each bout for further analysis.

For the optogenetic manipulation during different feeding phase experiments in Fig. 5D–F, the blue light (473 nm) was delivered at 20 Hz (20 ms pulse duration) during approaching or consumption phase, while the yellow light (593 nm) was applied constantly during approaching or consumption phase.

#### Open field test

On the day of the behavioral test, the mice were transferred to the testing room and were habituated to the room conditions for 3 h before the experiments started. The apparatus was cleaned with 20% ethanol to eliminate odor from other mice. The mice were placed in a plastic open field chamber (40 × 40 cm). The mice locations were monitored using custom MATLAB tracking software at 30-Hz sampling frequency. The chamber was conceptually divided into a central field (25 × 25 cm) and a peripheral field for analysis. The total locomotion and the ratio of time spent in the center were analyzed.

For acute chemogenetic activation experiments, mice were divided into two groups randomly and were treated with saline or CNO (2 mg/kg body weight in saline, IP) 30 min before testing. Mice were allowed to freely explore for 10 min. On the next day, mice were treated with the CNO or saline conversely and tested again. For chronic chemogenetic activation experiments, mice were not treated with CNO before test.

For optogenetic inhibition experiments, mice were placed in the chamber and yellow light stimulation (593 nm, continuous light with 5 min ON and 5 min OFF interleaved) were delivered for 20 minutes.

### Body weight and physiological parameters measurements

#### Body weight measurement

After virus injection, the mice were housed in groups (3–5 animals per cage) fed with the standard chow. After 3 weeks, the body weigh was measured daily. For TeNT-induced synaptic inactivation experiments, mice were fed with the standard chow or changed into high-fat food. For chemogenetic activation experiments, the different groups were fed with standard chow or changed to high-fat food. CNO (1 mg/kg in saline, IP) was injected twice per day.

#### Energy expenditure measurement

Energy expenditure was measured using an indirect calorimetry system (Oxymax, Columbus Instruments) installed under a constant environmental temperature (22–25 °C) and a 12-h light, 12-h dark cycle. Mice in each chamber had free access to food and water.

#### Blood-glucose measurement

To measure blood glucose, mouse tail was cut horizontally at the end with a razor blade and a small drop of blood was collected. Glucose levels were then measured using a blood glucose meter (Bayer). For chemogenetic activation experiments, basal blood glucose levels were first measured at the onset of the dark phase of the photoperiod. Following injections of CNO (2 mg/kg body weight in saline, IP), blood glucose levels were measured 30 minutes later. No food was supplied during the blood glucose measurement period. For TeNT-induced synaptic inactivation experiments, fasting blood-glucose were measured following an overnight fast (16 h). Then these fasted mice received an oral dose (1.5 g/kg) of 30% D-glucose solution (Catalog No. G6125, Sigma-Aldrich) by gavage. Blood was collected at baseline and 10, 30, 60, 90 and 120 min after glucose administration.

#### Blood pressure and heart rate measurement

For blood pressure and heart rate measurement, the BP-2000 Blood Pressure Analysis System™ (Visitech system) was employed and the user’s guide was followed. The BP-2000 uses transmission photoplethysmography, in which variations in the amount of light transmitted through the tail was analyzed to determine the blood pressure and pulse rate. The animals were first habituated to blood pressure measurement for 3 days prior to starting formal experiment. The measurements were performed at the 2–4 pm of each day. Mice were transferred to a quiet measuring room one to two hours before making measurements. Mice were handled gently in order to keep them as calm as possible. Place the animal inside a holder, with the cuff around their tails, and put the tail at the bottom of the V-shaped groove in the sensor. In order to allow the animals to warm up sufficiently to produce a good blood flow in the tail and allow them to get accustomed to being in the specimen holders, a minimum of 5 preliminary measurements was performed before the actual measurements begin in each session. Ten to twenty actual measurements were performed in each session.

For chemogenetic activation experiments, basal blood pressure and heart rate were measured following 3 days habituation. At the same time the next day, blood pressure and heart rate were measured again following injections of CNO (2 mg/kg body weight in saline, IP).

#### Rectal temperature measurement

To measure rectal temperature, a thermometer (TH-5 Thermalert, Physitemp) was used. Firstly, put petroleum jelly on the end of the probe of the thermometer. During the rectal temperature acquisition, the base of the tail of mouse was fixed with two fingers and then gently lifted while the animal gripped a metal rod on the cage lid with its front paws, thus allowing for exposure of the anogenital area. The excrement and urine was cleaned to minimize confounding effects by urination or defecation. Then the probe with petroleum jelly on it was inserted into the anal canal for 1.5 centimeter and the temperature was read when it stabilizes. The rectal temperature were measured at least for three consecutive days before the formal data were collected. For chemogenetic experiments, the rectal temperature was measured 30 minutes after the injection of CNO (Enzo, Catalog No. BML-NS105-0025, 2 mg/kg body weight in saline, IP).

#### Fiber photometry

Fiber photometry experiments were performed at least 3 weeks after AAV-GCaMP6s injection. The implanted fiber was connected to Fiber Optic Meter (ThinkerTech, Nanjing, China) through an optical fiber patch cord (200 μm, 0.37 NA, Inper, Hangzhou, China). To record fluorescence signals, a beam from a 480 LED was reflected with a dichroic mirror, focused with a lens coupled to a CMOS detector (Thorlabs, Inc. DCC3240M). The LED power at the tip of the patch cord was less than 50 μW.

LS^Nts^ neuronal calcium activity was recorded from mice infected with GCaMP6s during cue-conditioned or self-paced feeding. The licking signals were collected through a home-made lickmeter and acquired by the Fiber Optic Meter. Analysis of the signal was performed with custom-written MATLAB codes. The fluorescence change (ΔF/F) was calculated as (F-F0)/F0, where F0 is the baseline fluorescence signal (−6 to −4 s before the first lick or cue onset). The area under the curve (AUC) was calculated as the mean ΔF/F of the event, which were −6 to 0 s for the food-approach phase and 0 to 8 s for the food consumption phase.

To record Ca^2+^ from LS^Nts^→TU or LS^Nts^→SUM, AAV-Retro-DIO-GCaMP6s were injected to TU or SUM and optic fiber was implanted at LS in Nts-ires-Cre mice. In order to eliminate the effects of baseline drift, the raw fluorescence trace was corrected as described by Xiao et al. (Xiao et al., 2020). After baseline drift correction, the fluorescence signals were z-scored relative to the mean and standard deviation of the signals in a time window −6 to −4 s before the first lick onset.

#### Calcium imaging with miniature microscopy

Mice with baseplate and attached miniature microscope (UCLA miniscope V4, Open Ephys) were habituated to the imaging behavior chamber (35 cm × 30 cm) for at least three days before the imaging sessions. Imaging data was acquired at 30 Hz frame rate and with LED power as 15%–35%. Calcium imaging data was collected by UCLA Miniscope-DAQ-DT-Software. The synchronization between the miniature microscope and behavior-related events (lick and pump events) was achieved by a custom Arduino board.

Analysis of calcium imaging times series was performed in Python and MATLAB. The imaging data were spatially down-sampled (twofold in x-y) and temporally down-sampled (fourfold). Constrained non-negative matrix factorization (CNMF-E) for microendoscopy was used to extract GCaMP fluorescence responses associated with individual neurons from the processed data (Zhou et al., 2018). Briefly, we estimated individual neurons response by CNMF-E, and manually removed the obvious non-neural objects that had usually unrealistically small (1–5 pixels) or large (above 100 pixels) soma sizes.

In conventional calcium imaging, fluorescence signals are compared as ΔF/F. We used the inspected raw output of CNME-F as ΔF for the subsequent analysis. To report average fluorescence responses across neurons within a session, normalized ΔF was calculated as (ΔF − ΔF_baseline_)/ (ΔF_max_ − ΔF_min_). ΔF_baseline_ was the −6 to −4 s mean responses of a trial.

The Ca^2+^ response half width and bout duration were first estimated from linear curve fitting in MATLAB. The correlation coefficient between the response half width and bout duration was calculated with MATLAB function ‘corrcoef’.

To compare responses to different foods within a session, ΔF for each neuron was averaged across multiple trials. Then the z-score was calculated from this average response.

To classify Ca^2+^ responses as either ‘excited’ or ‘inhibited’ during consumption, baseline value was first calculated form the average fluorescent signal of ROI from −6 to −4 sec of each trial. Peak response was calculated from the average fluorescent signal ±1 s around the peak of each trial. Statistical comparison (Wilcoxon signed-rank test) between baseline values and peak responses for all trials was performed. The cell was classified as excitatory response if *p* < 0.05 and had a positive peak response. The cell was classified as inhibitory response if *p* < 0.05 and had a negative peak response. The cell was classified as no response if *p* > 0.05.

### Electrophysiological Recordings

Procedures for preparing acute brain slices and performing whole-cell recordings with optogenetic stimulation were similar to those described previously (Zhu et al., 2016). Coronal 250–300 μm slices containing the LS or TU were prepared using a vibratome (VT-1000S, Leica) in an ice-cold choline-based solution containing (in mM) 110 choline chloride, 2.5 KCl, 0.5 CaCl_2_, 7 MgCl_2_, 1.3 NaH_2_PO_4_, 1.3 Na-ascorbate, 0.6 Na-pyruvate, 25 glucose and 25 NaHCO_3_, saturated with 95% O_2_ and 5% CO_2_. Slices were incubated in 32 °C oxygenated artificial cerebrospinal fluid (in mM: 125 NaCl, 2.5 KCl, 2 CaCl_2_, 1.3 MgCl_2_, 1.3NaH_2_PO_4_, 1.3 Na-ascorbate, 0.6 Na-pyruvate, 25 glucose and 25 NaHCO_3_) for at least 1 h before recording. Slices were transferred to a recording chamber and superfused with 2 ml min^−1^ artificial cerebrospinal fluid. Patch pipettes (2–5 MΩ) pulled from borosilicate glass (PG10150-4, World Precision Instruments) were filled with a Cs-based low Cl^–^ internal solution containing (in mM) 135 CsMeSO_3_, 10 HEPES, 1 EGTA, 3.3 QX-314, 4 Mg-ATP, 0.3 Na-GTP, 8 Na_2_-phosphocreatine, 290 mOsm kg^−1^, adjusted to pH 7.3 with CsOH. Whole-cell voltage-clamp recording was performed at room temperature with a Multiclamp 700B amplifier and a Digidata 1440 A (Molecular Devices). Data were sampled at 10 kHz and analyzed with Clampfit (Molecular Devices) or MATLAB (MathWorks). A blue light-emitting diode (470 nm, Thorlabs) controlled by digital commands from the Digidata 1440 A was used to deliver photostimulation. To record light-evoked IPSCs, a blue light pulse (473 nm, 1 ms, 0.5~2 mW) was delivered through an optic fiber to illuminate the entire field of view. The IPSCs were recorded at holding potential of 0 mV in the presence of CNQX (10 μM). To block IPSCs, picrotoxin was added into recording chamber through perfusion system and incubated for at least 5 min.

#### Histological procedures

For c-fos immunostaining experiment, mice were sacrificed 90 min after the start of feeding. For c-fos RNAScope in situ hybridization experiment, mice were sacrificed 30 min after the start of feeding. For the verification of chemogenetic activation experiment, CNO (2 mg/kg body weight in saline, IP) were given 0.5 h before sacrificing the animal. Mice were euthanized with an overdose of pentobarbital sodium and transcardially perfused with phosphate buffered saline (PBS, pH 7.4) followed by 4% paraformaldehyde (PFA) in PBS. Brains were post-fixed for 16–24 hours in 4% PFA in PBS and dehydrated for 24–48 hours in 30% sucrose until they sank to the bottom. Tissue was embedded in Tissue-Tek OCT compound (Sakura) on dry ice before sectioning. Brains were cut into 40-μm sections with a cryostat (Leica). Free-floating cryosections were collected in PBS. Brain sections were first washed in PBS (3 ×10 min), then blocked at room temperature with 10% normal goat serum (GS)/0.3% Triton X-100 (PBST) and then incubated with primary antibody (Rabbit Anti-c-fos, Cell signaling) for overnight at 4 °C. Brain sections were washed in PBST (3 × 10 min), followed by incubation for 2 h with fluorophore-conjugated secondary antibody (1:1000 in 5% GS PBST, Invitrogen) and finally counterstained with DAPI (1:3,000).

For In situ RNA hybridization, mouse brain was cut into 18-μm sections with a cryostat (Leica) and mounted onto SuperFrost Plus microscope slides. The probes targeting c-fos (Advanced Cell Diagnostics, # 316921-C3), Nts (Advanced Cell Diagnostics, # 420441), Penk (Advanced Cell Diagnostics, # 318761), Crhr2 (Advanced Cell Diagnostics, # 413201), vGAT (Advanced Cell Diagnostics, # 319191-C3) and vGluT2 (Advanced Cell Diagnostics, # 319171-C3) were designed and validated by Advanced Cell Diagnostics. RNAscope v2 Assay (Advanced Cell Diagnostics, #323100) was used for all FISH experiments according to the manufacture’s protocol (ref: Wang et al., 2002). Briefly, brain sections were dried at 39 °C for 2 hours, rinsed in 1x PBS, treated with 3% hydrongen peroxide in methone, TR buffer for 15 min, treated with RNAScope protease III for 15 min at 40 °C. The brain sections were incubated with mRNA probes for 2 hours at 40 °C. The specific signals were then amplified with multiplexed implication buffer and detected with TSA Plus Flucerence Kit (Advanced Cell Diagnostics, #322809).

Images were obtained using an Olympus Virtual Slide Microscope (VS120-S6-W), and analyzed by an individual blind to the identity of experimental groups. Cell counting was done with custom-written MATLAB software, as described below.

#### Cell counting

To count the number of c-fos^+^, Nts^+^, Penk^+^, Crhr^2+^ and CTB^+^ cells, we collected 40 μm coronal sections of target brain regions for each mouse. The images were acquired with slide scanner (Olympus Virtual Slide Microscope, VS120-S6-W) and then cell counting was performed with custom-written MATLAB software.

### Statistics

Statistical analyses were done using GraphPad Prism (GraphPad Software V8), or Matlab (2017a, Mathworks). No statistics were used to predetermine sample size.

## Results

### Brain-wide c-fos mapping reveals the activation of neurotensin-positive neurons in the LS induced by hedonic feeding

**Fig. 1:**
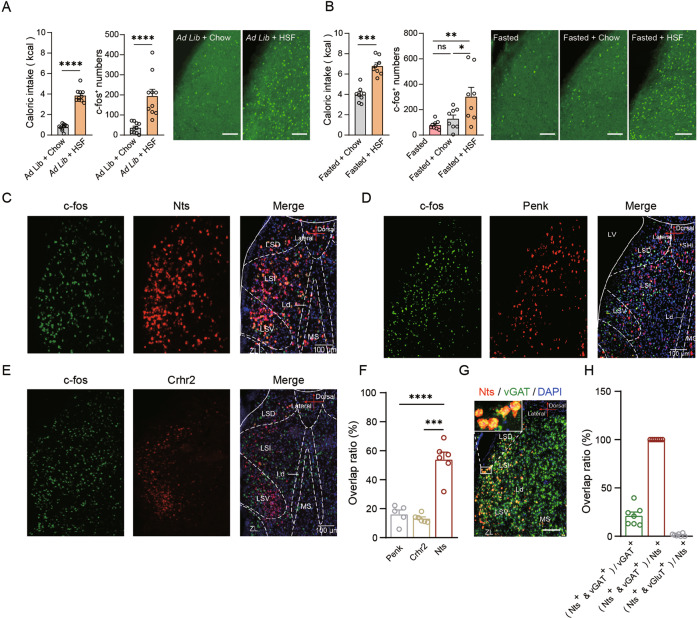
Nts-positive neurons in the LS were activated during hedonic feeding. **A** The LS was activated during the intake of high-sucrose food without an energy deficit. Left panel: Food intake measured in 2 h for control mice (*ad libitum* access to standard chow, *n* = 10) and hedonic feeding mice (intermittent access to high-sucrose food along with *ad libitum* access to standard chow, *n* = 10). Mann–Whitney U test. ****P < 0.0001. Middle panel: Number of c-fos^+^ neurons in the LS of control (*n* = 10) and hedonic feeding mice (*n* = 10). Mann–Whitney U test. ****P < 0.0001. Right panel: Representative images of c-fos immunostaining in the LS of control and hedonic-feeding mice. Scale bar: 100 μm. **B** The LS showed preferential activation when mice consumed high-sucrose food (HSF) compared with standard chow after fasting. Left panel: Food intake measured in 2 h for standard chow (*n* = 8) or high-sucrose food (*n* = 8) after an overnight fast. Mann–Whitney U test. ****P < 0.0001. Middle panel: Number of c-fos^+^ neurons in the LS of the fasted, fasted + chow and fasted + HSF groups. One-way ANOVA (F_(2, 21)_ = 6.558, *P* < 0.01) followed by Tukey’s post hoc test. ns, no significant difference, *P < 0.05, and **P < 0.01. Right panel: Representative images of c-fos immunostaining in the LS of the fasted, fasted + chow and fasted + HSF groups. Scale bar: 100 μm. **C** Representative images of double in situ hybridization experiments for c-fos (green) and Nts (red). Scale bar: 100 μm. **D** Representative images of double in situ hybridization experiments for c-fos (green) and Penk (red). Scale bar: 100 μm. **E** Representative images of double in situ hybridization experiments for c-fos (green) and Crhr2 (red). Scale bar: 100 μm. **F** Percentages of Penk^+^, Crhr2^+^ and Nts^+^ cells among the c-fos^+^ population: Penk^+^/c-fos^+^ = 19.7 ± 2.5%, Crhr2^+^/c-fos^+^ = 13.5 ± 1.3%, and Nts^+^/c-fos^+^ = 57.6 ± 2.5%. One-way ANOVA (F_(2,14)_ = 43.71, *P* < 0.01) followed by Tukey’s post hoc test. ***P < 0.001 and ****P < 0.0001. Means ± s.e.m. **G** Representative images of double in situ hybridization experiments for Nts (red) and vGAT (green). Scale bar: 100 μm. **H** Percentage of Nts^+^ cells in the vGAT^+^ population and percentage of vGAT^+^ cells in the Nts^+^ population: (Nts^+^ & vGAT^+^)/vGAT^+^ = 21.3 ± 3.8%, (Nts^+^ & vGAT^+^)/Nts^+^ = 100%, and (Nts^+^ & vGluT2^+^)/Nts^+^ = 1.38 ± 0.5%.

We employed a limited-access palatable food feeding protocol, which induced robust consumption of palatable food without fasting. This feeding protocol consisted of daily intermittent access to high-sucrose food for 2 hours, while standard laboratory chow food was always provided [15, 17]. Mice developed stable food intake in this 2-h period, while control mice with ad libitum access to standard chow ate little during this period (Fig. 1A and Fig. S1A, B). Food intake was primarily restricted to palatable food (Fig. S1C), suggesting that feeding was mainly driven by the palatability of the food—a hallmark of hedonic feeding.

We performed immunostaining for c-fos, a marker of recent neuronal activity, to examine hedonic feeding-activated neurons across the entire brain. Multiple brain regions were activated by hedonic feeding, including the paraventricular nucleus of the thalamus (PVT), LS, cingulate cortex (Cg), SUM, insula, hippocampus (Hipp), periaqueductal gray (PAG), ventral tegmental area (VTA), ventral lateral geniculate nucleus (VLGMC), TU and zona incerta (ZI) (Fig. S1D and Fig. 1A). Hypothalamic nuclei, including the paraventricular nucleus of the hypothalamus (PVN) and lateral hypothalamic area (LHA), showed increased c-fos expression, consistent with their roles in regulating feeding (Fig. S1D). Among them, the PVT and LS showed the highest c-fos expression (Fig. S1D).

Neurons involved in hedonic feeding should be more active when animals are consuming palatable food. We then compared the c-fos expression levels in the PVT and LS of mice consuming palatable food vs. regular chow after an overnight fast. The PVT showed similar levels of c-fos expression when mice consumed high-sucrose food and regular chow, whereas the LS showed preferential activation after mice consumed palatable food (Fig. 1B and Fig. S1E, F). Thus, the LS might play a unique role in regulating hedonic feeding.

The LS contains various neuronal populations defined by distinct molecular markers. To determine the molecular identity of LS neurons that were activated by hedonic feeding, we performed RNAScope double fluorescence in situ hybridization (dFISH) for the mRNAs encoding c-fos and the LS neuronal markers neurotensin (Nts), proenkephalin (Penk) and corticotropin-releasing factor (Crhr2). RNAScope revealed that 57.6% ± 2.5% of the hedonic feeding-activated LS neurons were Nts-positive, whereas only 19.7% ± 2.5% were Penk-positive and 13.5% ± 1.3% were Crhr2-positive (Fig. 1C–F). The hedonic feeding-activated c-fos^+^ cells were distributed from the rostral to caudal LS, which is similar to the expression pattern of Nts (Fig. S1G, H). We also performed dFISH with probes for neurotensin and the vesicular GABA transporter (vGAT), a marker of GABAergic neurons, or the vesicular glutamate transporter 2 (vGluT2), a marker of glutamatergic neurons in the septal area. Approximately all Nts-positive neurons were also vGAT-positive, while Nts-positive neurons constituted ~21% of GABAergic neurons in the LS (Fig. 1G, H).

### Silencing LS^Nts^ neurons promotes hedonic feeding of palatable food

**Fig. 2:**
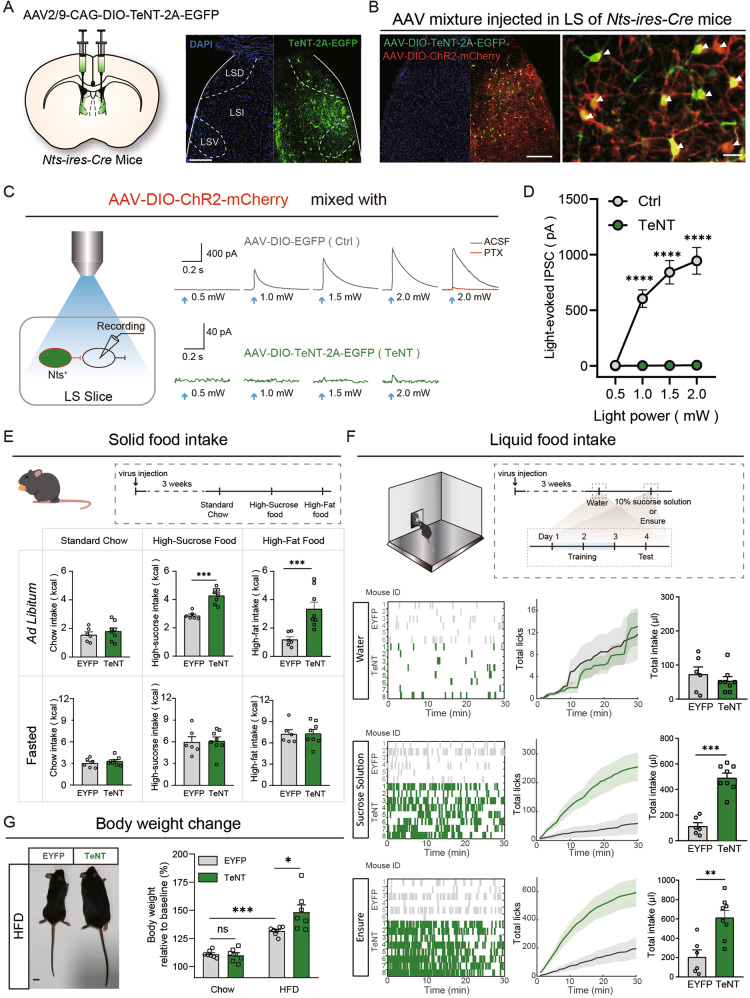
Silencing of LS^Nts^ neurons promotes hedonic feeding with palatable food. **A** Left panel: Schematic showing the injection of AAV-DIO-TeNT into the LS for the synaptic silencing of LS^Nts^ neurons. Right panel: representative image of the injection site and viral expression in the LS of *Nts-ires-Cre* mice. Scale bar: 500 μm. **B** Representative image showing the expression of ChR2 (red) and TeNT (green) in LS^Nts^ neurons. Scale bar: 500 μm (upper panel), 20 μm (lower panel). **C** Schematic showing the experiment used to record postsynaptic currents in LS slices induced by optogenetic stimulation of LS^Nts^ neurons. In the slice in which ChR2 was coexpressed with EYFP, blue light stimulation evoked robust IPSCs (gray trace), which was blocked by picrotoxin (red trace). In the slice in which ChR2 was coexpressed with TeNT, blue light stimulation failed to evoke IPSCs (green trace). **D** Statistics for the amplitudes of light-evoked IPSCs in TeNT-expressing (*n* = 6 cells) and EYFP-expressing (*n* = 7 cells) mice. Two-way ANOVA (F_(1,11)_ = 53.46, *P* < 0.0001) followed by Sidak’s post hoc test. ***P < 0.0001. Means ± s.e.m. **E** Quantification of 2-h solid food intake in EYFP- (gray bar, *n* = 6) and TeNT-expressing mice (green bar, *n* = 8). Upper panel: TeNT expression increased the intake of high-sucrose and high-fat food but not standard chow under ad libitum conditions. Lower panel: TeNT expression had no effect on food intake under fasted conditions. Mann–Whitney U test. ***P < 0.001. Means ± s.e.m. **F** Quantification of 2-h liquid food intake by EYFP- (gray bar, *n* = 6) and TeNT-expressing mice (green bar, *n* = 8). TeNT expression increased the intake of palatable liquid food but not water. Representative licking behavior (left panel), cumulative licks (middle panel) and total intake (right panel) of water (upper panel), sucrose solution (middle panel) and Ensure (lower panel) in EYFP- (gray, *n* = 6) and TeNT-expressing mice (green, *n* = 8). Mann–Whitney U test. **P < 0.01 and ****P < 0.0001. Means ± s.e.m. **G** Quantification of changes in the body weights of EYFP- (gray bar, *n* = 7) and TeNT-expressing (green bar, *n* = 7) mice. Left panel: Representative images showing the EYFP- and TeNT-expressing mice after 6 weeks of feeding on a high-fat diet. Right panel: TeNT expression promoted increased body weight of mice fed the high-fat diet, but not standard chow. Mann–Whitney U test. ns, no significant difference, *P < 0.01, and ***P < 0.001. Means ± s.e.m.

We employed a Cre-dependent adeno-associated virus (AAV)-mediated expression strategy to specifically manipulate the activity of LS^Nts^ neurons and study the function of LS^Nts^ neurons in hedonic feeding. By injecting AAVs carrying double-floxed EGFP (AAV-DIO-EGFP) into the LS of *Nts-ires-Cre* mice [18], we confirmed that the expression was restricted to Nts-positive neurons in the LS (Fig. S2A, B). We then employed tetanus neurotoxin (TeNT), a protease that blocks neurotransmitter release by cleaving synaptobrevin-2, which is widely used as a molecular tool for synaptic silencing [19]. To verify the efficiency of TeNT-induced synaptic silencing, we injected Cre-dependent AAVs expressing channelrhodopsin-2 (ChR2) together with enhanced green-fluorescent protein (EGFP) as a control or tetanus neurotoxin (TeNT) for synaptic silencing into the LS of *Nts-ires-Cre* mice (Fig. 2B). In LS slices prepared from EGFP-expressing control mice, light stimulation of ChR2-expressing LS^Nts^ neurons evoked robust picrotoxin-sensitive inhibitory postsynaptic currents (IPSCs) in all neighboring ChR2-negative LS neurons (7/7) (Fig. 2C, D). The expression of TeNT almost completely blocked synaptic transmission from LS^Nts^ neurons (Fig. 2C, D). Strikingly, silencing of the synaptic outputs from LS^Nts^ neurons strongly promoted the consumption of palatable high-sucrose and high-fat food but not standard chow under *ad libitum* conditions (Fig. 2E, upper panel). This orexigenic effect was observed in both the 2 and 24-h feeding periods (Fig. 2E and Fig. S3A–C). However, in fasted mice, when homeostatic needs became the major driver of feeding, TeNT expression had no effect on food intake, regardless of the food type (Fig. 2E, lower panel). We also examined the effect of TeNT expression on the intake of liquid food during free delivery of a small, fixed volume (10 μl) of a palatable sucrose solution or Ensure using a motorized lick spout that was equipped with a lickometer (Fig. S1I–K). TeNT expression also significantly promoted the intake of palatable liquid food, both sucrose solution and Ensure, but not water (Fig. 2F). We also employed an optogenetic approach, which had superior temporal precision, to silence LS^Nts^ neurons. Consistent with TeNT-mediated synaptic silencing, optogenetic silencing of LS^Nts^ neurons substantially increased the intake of palatable Ensure, which occurred immediately after light on (Fig. S3G–K). Together, these results reveal an important role for LS^Nts^ neurons in regulating hedonic feeding.

The robust effect of LS^Nts^ silencing on palatable food intake suggests that the energy balance might be disturbed; we then examined locomotor activity and energy expenditure. Optogenetic-mediated neuronal inhibition or TeNT-mediated synaptic silencing of LS^Nts^ had no effect on the general locomotor activity of mice in the open field test (Figs. S2G and 3D). TeNT expression did not change the energy expenditure of mice in metabolic cages (Fig. S3E, F). Consistent with these results, TeNT expression significantly increased the body weight of mice that were fed a high-fat diet for 3 weeks (Fig. 2G). However, this increase in body weight was not observed in mice fed regular chow (Fig. 2G). These results confirm the critical role of LS^Nts^ neurons in regulating hedonic feeding and body weight.

### Activation of LS^Nts^ neurons suppresses overall feeding

**Fig. 3:**
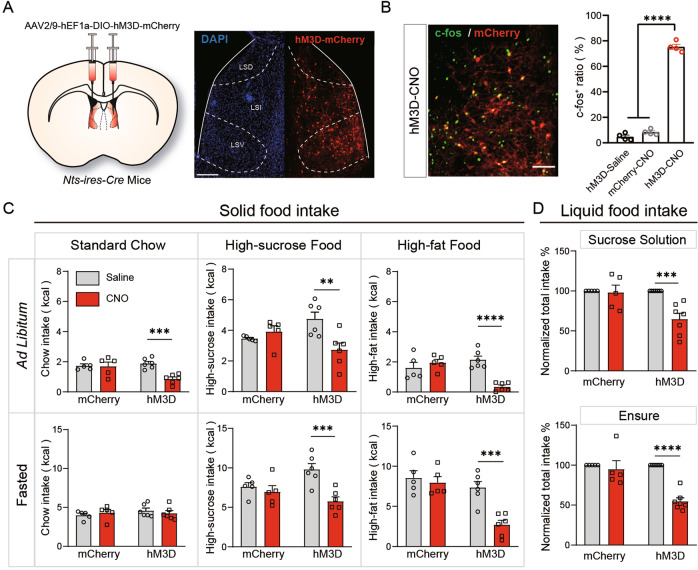
Activation of LS^Nts^ neurons suppresses overall feeding. **A** Left panel: Schematic showing the injection of AAV-DIO-hM3D into the LS for chemogenetic activation of LS^Nts^ neurons. Right panel: Representative image of the injection site and viral expression in the LS of *Nts-ires-Cre* mice. Scale bar: 500 μm. **B** Left panel: Representative image showing that the CNO injection (2 mg/kg) induced robust c-fos expression of LS^Nts^ neurons in hM3D-expressing mice. Scale bar: 100 μm. Right panel: Percentage of c-fos^+^ cells among LS^Nts^ neurons from the “hM3D + saline” (*n* = 4), “EYFP + CNO” (*n* = 4) and “hM3D + CNO” (*n* = 4) groups. One-way ANOVA (F_(2,9)_ = 684.5, *P* < 0.001) followed by Dunnett’s post hoc test. ****P < 0.0001. **C** Quantification of 2-h solid food intake after saline (gray bar) and CNO (red bar) injection in mCherry- and hM3D-expressing mice. Upper panel: CNO injection reduced food intake under ad libitum conditions in hM3D-expressing (*n* = 6) but not mCherry-expressing mice (*n* = 5). Two-way ANOVA (standard chow, F_(1,18)_ = 8.202, *P* < 0.05; high-sucrose food, F_(1,18)_ = 9.941, *P* < 0.01; high-fat food, F_(1,18)_ = 19.27, *P* < 0.001) followed by Sidak’s post hoc test. **P < 0.01, ***P < 0.001, and ****P < 0.0001. Lower panel: CNO injection reduced high-sucrose and high-fat food intake under fasted-refed conditions in hM3D-expressing (*n* = 6) but not mCherry-expressing mice (*n* = 5). Two-way ANOVA (standard chow, F_(1,18)_ = 0.9784, *P* > 0.05; high-sucrose food, F_(1,18)_ = 5.234, *P* < 0.05; high-fat food, F_(1,18)_ = 6.420, *P* < 0.05) followed by Sidak’s post hoc test, ***P < 0.001, means ± s.e.m. **D** CNO injection reduced the total intake of sucrose solution (upper panel) and Ensure (lower panel) by hM3D-expressing (*n* = 7) but not mCherry-expressing mice (*n* = 5). Sucrose solution: two-way ANOVA (F_(1,20)_ = 7.96, *P* < 0.05) followed by Sidak’s post hoc test. ***P < 0.001. Ensure: two-way ANOVA (F_(1,20)_ = 15.70, *P* < 0.001) followed by Tukey’s post hoc test. ****P < 0.0001. Means ± s.e.m.

We next examined the effects of the activation of LS^Nts^ neurons on food intake. We injected Cre-dependent AAVs expressing the excitatory designer receptors exclusively activated by designer drugs (DREADDs) hM3D [20] or mCherry into the LS of *Nts-ires-Cre* mice (Fig. 3A). Three weeks later, an intraperitoneal (IP) injection of clozapine N-oxide (CNO, 2 mg/kg), but not saline, resulted in robust c-fos expression in hM3D-expressing LS neurons (Fig. 3B). Under *ad libitum* conditions, chemogenetic activation of LS^Nts^ neurons robustly decreased food intake, irrespective of the food type (Fig. 3C, upper panel). However, in the fasted condition, activation of LS^Nts^ neurons only decreased the intake of palatable high-fat and high-sucrose food but not standard chow (Fig. 3C, lower panel). Activation of LS^Nts^ neurons also suppressed the intake of palatable liquid food, both the sucrose solution and Ensure (Fig. 3D–E). Chemogenetic activation of LS^Nts^ had no effect on the general locomotor activity of the mice (Fig. S2D). Manipulating the activity of LS^Nts^ neurons had no effect on anxiety-related behavior in the open field test (Figs. S2E and 2H) and had no effect on blood pressure, body temperature, blood glucose level or heart rate (Fig. S4), suggesting a specific role for LS^Nts^ neurons in regulating feeding.

### GABA and neurotensin signaling differentially regulate hedonic and overall feeding

Our results showed that silencing LS^Nts^ neurons specifically promoted hedonic feeding, while activation of LS^Nts^ neurons suppressed overall feeding. We wondered whether different signals from LS^Nts^ neurons might differentially regulate hedonic-specific and overall feeding. At the basal physiological activity level, GABA was released and acted on postsynaptic neurons, as we recorded picrotoxin-sensitive IPSCs in local postsynaptic neurons following brief optogenetic stimulation (1 ms) of LS^Nts^ (Fig. 2C). Upon strong chemogenetic activation, the neurotensin peptide has been detected in downstream regions after a CNO injection (Patterson CM et al., 2015). We thus hypothesized that GABA release at the basal activity level suppresses hedonic feeding, while neurotensin was further recruited upon strong LS^Nts^ activation to suppress overall feeding.

**Fig. 4:**
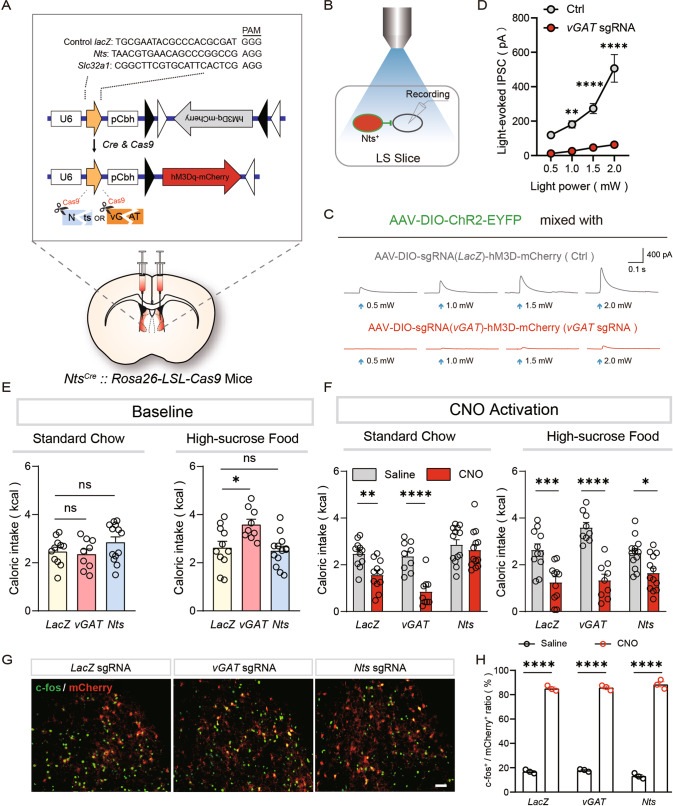
GABA and neurotensin signaling differentially regulate hedonic and overall feeding. **A** Schematic showing the experimental design for CRISPR/Cas9-mediated knockdown of vGAT and Nts. The AAV encoding vGAT-targeting, Nts-targeting, or control LacZ-targeting sgRNA and Cre-inducible hM3D was injected into *Nts-ires-Cre* mice crossed with Cre-inducible Cas9 knock in mice. **B** Schematic showing the experimental design for the verification of vGAT knockdown. In LacZ control or vGAT knockdown mice, ChR2 was expressed in LS^Nts^ neurons, and whole-cell patch clamp recordings were performed on neighboring Nts-negative neurons. **C** Representative traces of light-evoked IPSCs for LacZ control (gray) and vGAT knockdown (red) mice at different light powers (from left to right: 0.5 mW, 1.0 mW, 1.5 mW, 2.0 mW). **D** Statistics for the amplitudes of light-evoked IPSCs in the LacZ control (gray, *n* = 8 cells) and vGAT knockdown (red, *n* = 8 cells) mice. Two-way ANOVA (F_(1,14)_ = 50.87, *P* < 0.0001) followed by Sidak’s post hoc test. **P < 0.01 and ***P < 0.0001, means ± s.e.m. **E** Baseline food intake (left panel: standard chow, right panel: high-sucrose food) by LacZ control (yellow, *n* = 11), vGAT knockdown (red, *n* = 9) and Nts knockdown (blue, *n* = 13) mice. One-way ANOVA (standard chow, F_(2,230)_ = 1.716, *P* > 0.05; palatable food, F_(2,30)_ = 6.563, *P* < 0.01) followed by Tukey’s post hoc test. ns, no significant difference and *P < 0.05. Means ± s.e.m. **F** Effects of chemogenetic activation of LS^Nts^ neurons on food intake (left panel: standard chow, right panel: palatable food) by LacZ control (*n* = 11), vGAT knockdown (*n* = 9) and Nts knockdown (*n* = 13) mice. Two-way ANOVA (standard chow, F_(2,60)_ = 4.661, *P* < 0.05; palatable food, F_(2,60)_ = 5.583, *P* < 0.01) followed by Sidak’s post hoc test. ns, no significant difference, *P < 0.05, ***P < 0.001, and ****P < 0.0001, means ± s.e.m. **G** Representative images showing that the CNO (2 mg/kg) injection induced robust c-fos expression in LS^Nts^ neurons in LacZ control, vGAT knockdown and Nts knockdown mice. Scale bar: 100 μm. **H** Statistical analysis of the ratio of c-fos^+^ cells after saline and CNO injection in LacZ control (*n* = 3), vGAT knockdown (*n* = 3) and Nts knockdown (*n* = 3) mice. One-way ANOVA (F_(5,12)_ = 854.6, *P* < 0.0001) followed by Tukey’s post hoc test. ****P < 0.0001. Means ± s.e.m.

To examine this hypothesis, we used CRISPR/Cas9-mediated genome editing [21] to knock down GABA or neurotensin release in LS^Nts^ neurons. We designed a single guide RNA (sgRNA) targeting vesicular GABA transporter (vGAT), which is responsible for packing GABA into synaptic vesicles and is indispensable for GABAergic synaptic transmission [22], to reduce GABA release. After crossing *Nts-ires-Cre* mice with Cre-inducible Cas9 knock-in mice [21], we injected an AAV carrying both vGAT-targeting (sgRNA) and Cre-inducible hM3D-mCherry into the LS to express hM3D in vGAT-knockdown cells (Fig. 4A). To verify the efficiency of vGAT knockdown, we also injected AAV-DIO-ChR2 into the LS of vGAT knockdown animals and recorded IPSCs from postsynaptic neurons. Brief blue light stimulation (1 ms) evoked robust IPSCs in all recorded neurons (8/8) from control LacZ sgRNA animals (Fig. 4B–D, gray line). However, the same light stimulation failed to evoke any IPSCs in all recorded neurons (0/8) from vGAT knockdown animals (Fig. 4C, D, red line). These results demonstrated the high efficiency of our CRISPR/Cas9 strategy to disrupt GABA signaling.

vGAT knockdown in LS^Nts^ neurons had no effect on the intake of standard chow but significantly increased the intake of palatable high-sucrose food (Fig. 4E), suggesting that GABA specifically suppresses hedonic feeding under basal condition. Using a similar approach, we designed an Nts-targeting sgRNA to knock down the neurotensin peptide. Neurotensin knockdown had no effect on food intake, regardless of the food type (Fig. 4E). These results suggest that under basal physiological condition, GABA, but not neurotensin, signaling mediates the suppressive effect of LS^Nts^ neurons on hedonic feeding.

Next, we examined the effect of vGAT or neurotensin knockdown upon chemogenetic activation of LS^Nts^ neurons. Chemogenetic activation of LS^Nts^ by a CNO injection induced robust c-fos expression in all three groups of mice (Fig. 4G, H). In LacZ control mice, chemogenetic activation of LS^Nts^ neurons suppressed food intake, irrespective of the food type (Fig. 4F), consistent with our previous result. In vGAT knockdown mice, the administration of CNO also significantly suppressed food intake (Fig. 4F), suggesting that GABAergic transmission is not required when LS^Nts^ neurons were strongly activated. In Nts knockdown mice, the CNO injection still suppressed the intake of palatable food but failed to suppress chow intake, further indicating a specific role for GABA signaling in suppressing hedonic feeding. (Fig. 4F).

Taken together, these CRISPR/Cas9 knockdown experiments indicate that both GABA and neurotensin signaling from LS^Nts^ neurons regulate feeding, but they act at different activity levels. GABA released from LS^Nts^ neurons provides tonic inhibition to suppress hedonic feeding at the basal level, while neurotensin is further recruited upon strong activation to suppress overall feeding.

### Population LS^Nts^ activity exhibits biphasic responses associated with food-seeking and consumption behaviors

We employed fiber photometry [23] to assess the dynamics of LS^Nts^ neuronal activity in feeding behavior and the involvement of these neurons in hedonic feeding. We transduced LS^Nts^ neurons with a Cre-dependent AAV expressing a genetically encoded Ca^2+^ indicator (GCaMP6s) [24] and implanted optic fibers into the LS of *Nts-ires-Cre* mice. Using fiber photometry, we recorded population Ca^2+^ signals from LS^Nts^ neurons during food seeking and consumption. We first examined the activity of LS^Nts^ neurons during cue-conditioned delivery of a small, fixed volume of palatable Ensure (Fig. S5A). LS^Nts^ neurons showed robust Ca^2+^ dynamics during feeding (Fig. S5B). However, the activity was not modulated by food-predictive auditory cues or food delivery when the Ca^2+^ signal was aligned to the cue onset (Fig. S5C). In contrast, when the Ca^2+^ signal was aligned to the first lick after cue, we observed an increase in LS^Nts^ activity before the first lick when the animal approached the food spout. This increased activity was followed by a more robust decrease in activity when animals started to consume the food (Fig. S5D).

**Fig. 5:**
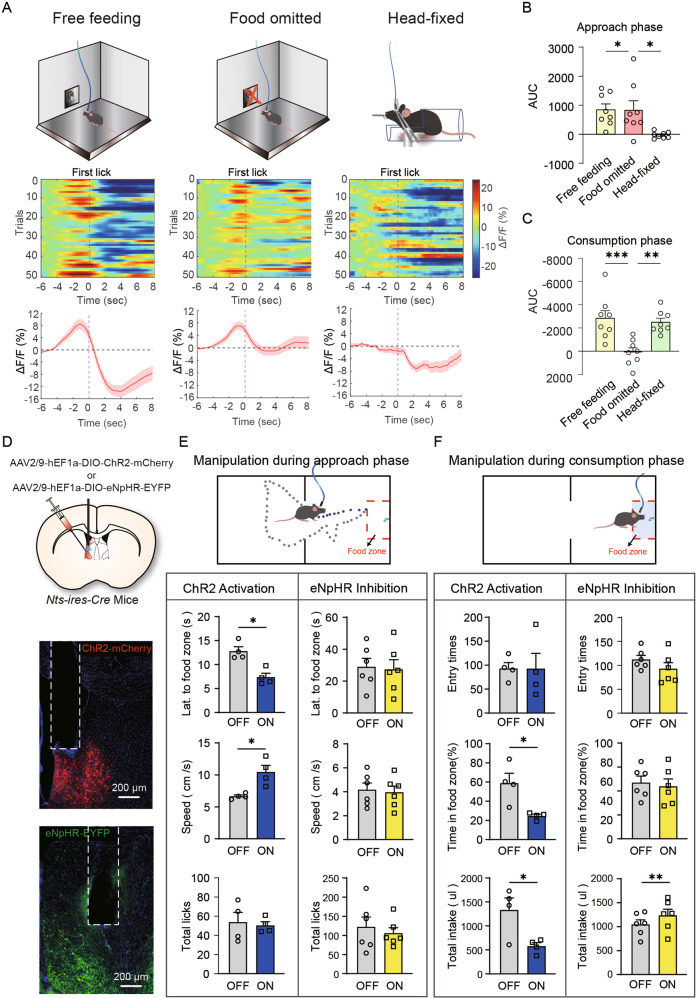
The biphasic activity of LS^Nts^ neurons drives food seeking and consumption. **A** Left panel: Population Ca^2+^ activity of LS^Nts^ neurons during free feeding of Ensure. Middle panel: Population Ca^2+^ activity of LS^Nts^ neurons when food was omitted. Right panel: Population Ca^2+^ activity of LS^Nts^ neurons when the animal was head-fixed and fed Ensure. Dashed vertical line: first lick. **B** Area under the curve of Ca^2+^ activity during the food approach phase (*n* = 8). One-way ANOVA (F_(2, 21)_ = 5.49, *P* < 0.01) followed by Tukey’s post hoc test. *P < 0.05. Means ± s.e.m. **C** Area under the curve of Ca^2+^ activity during the food consumption phase (*n* = 8). One-way ANOVA (F_(2, 21)_ = 11.05, *P* < 0.01) followed by Tukey’s post hoc test. **P < 0.01 and ***P < 0.001. Means ± s.e.m. **D** Upper panel: Schematic showing the injection of AAV-DIO-ChR2 or AAV-DIO-eNpHR into the LS for optogenetic manipulation of LS^Nts^ neurons. Middle panel: Representative image showing the expression of ChR2-mCherry in LS^Nts^ neurons. Lower panel: Representative image showing the expression of eNpHR-EYFP. **E** The effect of optogenetic activation (blue, *n* = 4) or inhibition (yellow, *n* = 6) of LS^Nts^ neurons during the food approach phase on food seeking and food consumption. Wilcoxon signed-rank test. *P < 0.05. **F** The effect of optogenetic activation (blue, *n* = 4) or inhibition (yellow, *n* = 6) of LS^Nts^ neurons during the food consumption phase on food seeking and food consumption. Wilcoxon signed-rank test. *P < 0.05 and **P < 0.01.

We also recorded Ca^2+^ signals during a free-access, self-paced feeding protocol [25] in which auditory cues were absent and food spouts were always available. Consistent with cue-conditioned feeding, we observed biphasic LS^Nts^ neuronal Ca^2+^ dynamics that exhibited an excitatory response during the food-approach phase and an inhibitory response during the food-consumption phase (Fig. 5A, left panel). The excitatory response during the approach phase was observed only when the animal was fed palatable Ensure but not regular food or diluted Ensure (Fig. S6A–C), suggesting that this activity was not caused by running or other motor artifacts. However, the inhibitory response observed during the consumption phase was similar across different food types (Figs. S6A–C and 6E, F). Both excitatory and inhibitory responses diminished when food was replaced with water (Fig. S6D).

Based on these observations, we hypothesized that the excitatory responses of LS^Nts^ neurons during the approach phase might represent the motivation to obtain palatable food and were thus important to drive food seeking and approach behaviors, while the inhibitory responses during the consumption phase might be critical for overall consummatory behavior. We omitted food delivery in some trials to test this hypothesis and found that the inhibitory responses after the first lick disappeared, while the excitatory responses during the approach phase were preserved (Fig. 5A, middle panel). We also examined LS^Nts^ activity in a head-fixed body-restrained feeding procedure in which the opportunity to engage in food seeking was removed; we found that the excitatory responses before the first lick disappeared, but the inhibitory responses during consumption were preserved (Fig. 5A, right panel).

The biphasic responses predict that LS^Nts^ neuron activation during the approach phase would facilitate food seeking, while LS^Nts^ neuron inhibition during the consumption phase is required for food consumption. Our previous TeNT synaptic inactivation and chemogenetic activation did not distinguish between the approach-locked and consumption-locked neuronal dynamics that we measured in vivo. Taking advantage of the millisecond time resolution provided by the optogenetic method, we specifically manipulated the activity of LS^Nts^ neurons during the approach phase vs. the consumption phase and examined its effects on food seeking and food consumption.

We then expressed the excitatory opsin ChR2 [26] in LS^Nts^ neurons for optogenetic activation and examined the effect of temporally precise optogenetic activation during different feeding phases (Fig. 5D). Light stimulation during the food-approach phase significantly shortened the latency and increased the speed to the food zone (Fig. 5E), suggesting that activation of LS^Nts^ neurons during the approach phase facilitated food-seeking behavior. However, this optogenetic manipulation had no effect on food consumption (Fig. 5E). In contrast, optogenetic activation of LS^Nts^ neurons during the consumption phase profoundly suppressed food consumption, while it did not change the entry times into the food zone but decreased the time spent within the food zone (Fig. 5F). We next expressed inhibitory opsin eNpHR [27] in LS^Nts^ neurons to achieve temporally precise optogenetic silencing (Fig. 5D). Consistently, optogenetic inhibition of LS^Nts^ neurons during the approach phase had no effect on consummatory behavior, while optogenetic inhibition during the consumption phase strongly promoted total food intake (Fig. 5E, F).

### Miniature microscopic Ca^2+^ imaging reveals two populations of LS^Nts^ neurons that are activated or inhibited during feeding

**Fig. 6:**
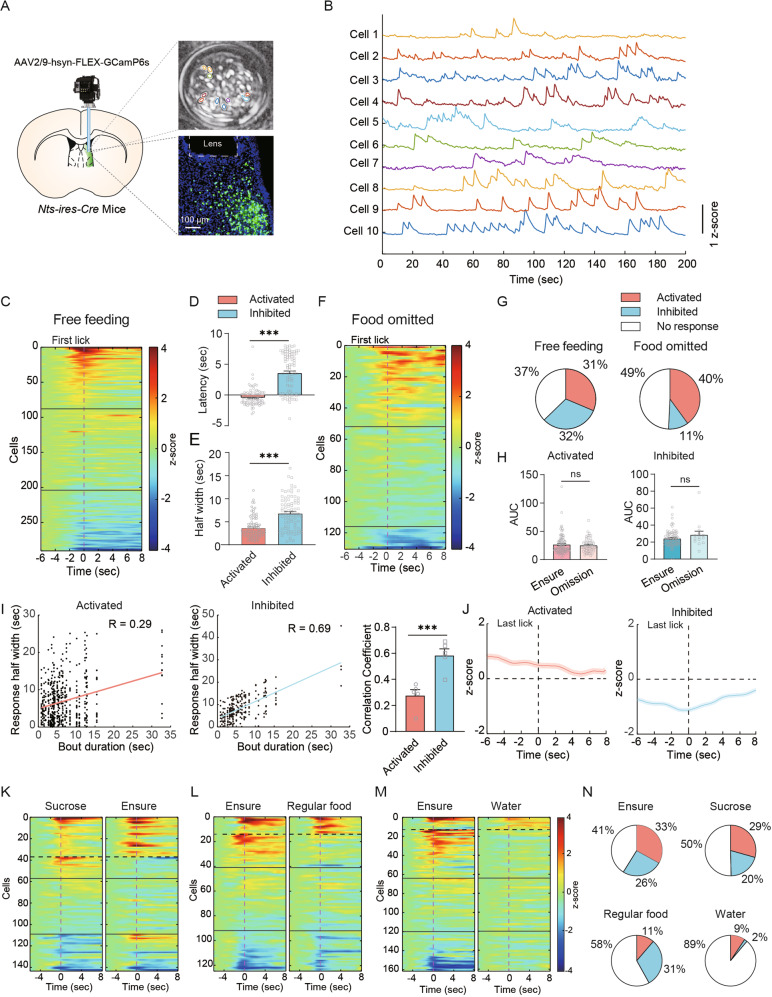
Miniscope Ca^2+^ imaging reveals two populations of LS^Nts^ neurons that are activated and inhibited during feeding. **A** Schematic showing Ca^2+^ imaging of individual LS^Nts^ neurons with a head-mounted miniature microscope. **B** Ca^2+^ dynamics of 10 representative LS^Nts^ cells during feeding. Scale bar: 1 z score. **C** Ca^2+^ responses of 290 LS^Nts^ cells aligned to the first lick during free feeding of Ensure. Approximately 31% of cells were activated, and 32% of cells were inhibited. **D** The time to the peak of activated cells (*n* = 88) was shorter than the time to the trough of inhibited cells (*n* = 86). Mann–Whitney U test. ***P < 0.001. **E** The response half-width of activated cells (*n* = 88) was shorter than that of inhibited cells (*n* = 86). Mann–Whitney U test. ***P < 0.001. F. Ca^2+^ responses of 130 LS^Nts^ cells aligned to the first lick under the food-deficient condition. Approximately 40% of cells were activated, and 11% of cells were inhibited. G. Percentages of activated, inhibited and nonresponsive cells under free feeding (left panel) and food omission (right panel) conditions. **H** Area under the curve of Ca^2+^ activity for activated (left panel) and inhibited (right panel) cells during the free feeding of Ensure and food omission trials. Mann–Whitney U test. Ns, no significant difference. I. Correlation between the response half-width and bout duration for inhibited (left panel) and activated neurons (middle panel). Right panel: The correlation coefficient between the response and behavior of inhibited cells was larger than that of activated cells. Mann–Whitney U test. ***P < 0.001. **J** Ca^2+^ response of activated (right panel) and inhibited (left panel) cells when aligned to the last lick during free feeding of Ensure. K. Ca^2+^ response of LS^Nts^ neurons to the sucrose solution and Ensure (*n* = 143 neurons from 4 mice) grouped by k-means clustering. Vertical dashed line: first lick. **L** Ca^2+^ response of LS^Nts^ neurons to Ensure and regular food (*n* = 124 neurons from 4 mice) grouped by k-means clustering. Vertical dashed line: first lick. **M** Ca^2+^ response of LS^Nts^ neurons to Ensure and water (*n* = 161 neurons from 4 mice) grouped by k-means clustering. Vertical dashed line: first lick. **N** Percentages of activated, inhibited and nonresponsive cells identified during the free feeding of Ensure, sucrose solution, regular food and water.

Because fiber photometry records summated Ca^2+^ activity from multiple LS^Nts^ neurons, two possible scenarios might explain the biphasic responses of LS^Nts^ neurons during feeding. First, distinct subpopulations of LS^Nts^ neurons were activated or inhibited. Second, a single population of LS^Nts^ neurons was first activated during food approach and then inhibited during food consumption. To distinguish these two possibilities, we performed in vivo Ca^2+^ imaging of individual LS^Nts^ neurons in freely moving mice using a head-mounted miniature microscope and an implanted gradient index (GRIN) lens [28] (Fig. 6A, B).

We expressed GCaMP6s in LS^Nts^ neurons and imaged the Ca^2+^ activity of individual cells through a head-mounted miniature microscope during free-access, self-paced feeding of palatable Ensure. LS^Nts^ neurons were most responsive when animals approached and started consuming food (around the first lick); 31% of neurons were significantly activated, and 32% were significantly inhibited (Fig. 6C, G). The activated neurons tended to respond faster; they reached their peak at 0.3 ± 0.1 sec before the first lick, while the inhibited cells reached their nadir at 3.6 ± 0.3 s after the first lick (Fig. 6D). The activated cells also showed a narrower response window, with a significantly smaller half width (3.7 ± 0.3 s) than the inhibited cells (6.8 ± 0.4 s) (Fig. 6E and Fig. S6G). These differences in the temporal kinetics of the two neuronal populations might contribute to the biphasic response that we observed in our fiber photometry recording. To test this possibility, we normalized the response of each cell and added them together; we found that the summed population response exhibited a biphasic response (Fig. S7H), which is reminiscent of the photometry response.

We examined the precise role of the activated and inhibited LS^Nts^ neuronal populations in feeding by performing imaging during food omission trials. When food was omitted, the percentage of inhibited LS^Nts^ neurons decreased to 11% (Fig. 6F, G), while the portion of activated neurons remained high (40%), suggesting that the inhibited neurons were primarily involved in the consummatory phase of feeding behaviors. The mean response amplitude of both activated and inhibited neurons under food-deprived conditions was similar to that under free feeding conditions (Fig. 6H). We next examined whether the response of inhibited neurons could track consummative behavior. We analyzed the relationship between the Ca^2+^ response and consumptive licking behavior in each trial and observed a significant correlation between bout duration and the response duration of the inhibited neuronal population (Fig. 6I). The correlation coefficient calculated between the bout duration and response half width was significantly higher for the inhibited population (0.69 ± 0.05) than for the activated population (0.28 ± 0.05) (Fig. 6I), suggesting a role for the inhibited LS^Nts^ population in consummative behavior. To further examine whether the inhibited neurons can terminate food consumption, we aligned responses to the last lick, and found that the response returned to baseline immediately after the last lick (Fig. 6J). Thus, inhibited LS^Nts^ neurons contribute primarily to the consumption of food.

Next, we monitored the activity of LS^Nts^ neurons during feeding of foods with different palatability. The foods tested included palatable Ensure or sucrose solution, neutral regular food or water. We delivered two different foods sequentially in the same session and used unsupervised k-means clustering of responses to two different foods to achieve the accurate identification of responses of the same cells to different foods [25]. We compared responses from free feeding of Ensure and sucrose solution and observed similar responses. Both Ensure and sucrose activated and inhibited a similar proportion of LS^Nts^ neurons (Fig. 6K). The LS^Nts^ neurons that were activated and inhibited by Ensure and sucrose were largely the same subpopulation. However, the proportion of activated cells was significantly lower during feeding of regular food (11%) or water (9%) (Fig. 6L–N). These results indicated that the activated LS^Nts^ neurons are more tightly correlated with the palatability of the food.

### LS^Nts^ neurons projecting to the tuberal nucleus (TU) specifically regulate hedonic feeding

**Fig. 7:**
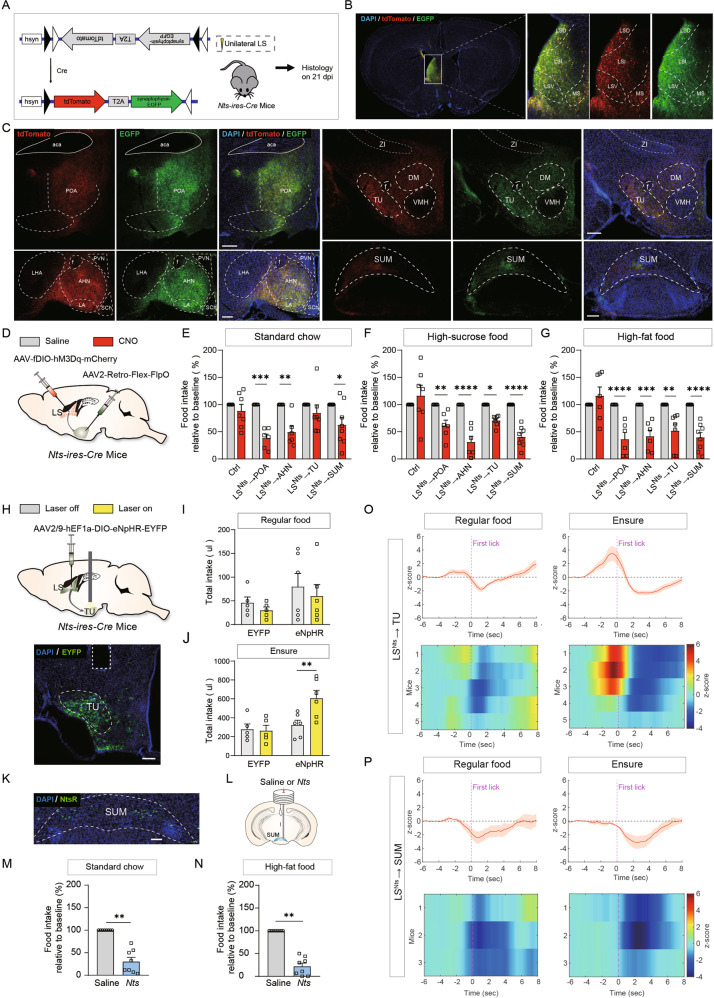
LS^Nts^ neurons project to the TU to regulate hedonic feeding. **A** Schematic showing the SynaptoTag AAV strategy to map the projections of LS^Nts^ neurons. **B** Representative image of the injection site and viral expression in the LS of *Nts-ires-Cre* mice. **C** Representative images showing tdTomato-expressing axons and GFP-expressing axon terminals in different regions. Scale bar: 200 μm. **D** Schematic showing the viral strategy used to activate LS^Nts^ neurons projecting to one specific downstream target through a chemogenetic approach. Quantification of the intake of standard chow (**E**), high-sucrose (**F**) and high-fat (**G**) food after chemogenetic activation of each LS^Nts^ projection. Control group, *n* = 7; LS^Nts^→POA group, *n* = 6; LS^Nts^→AHN group, *n* = 6; LS^Nts^→TU group, *n* = 7; LS^Nts^→SUM group, *n* = 8. Two-way ANOVA (standard chow, F_(4,68)_ = 2.854, *P* < 0.05; high-sucrose food, F_(4,58)_ = 9.145, *P* < 0.0001; high-fat food, F_(4,58)_ = 4.541, *P* < 0.0001) followed by Sidak’s post hoc test. *P < 0.05, **P < 0.01, ***P < 0.001, and ****P < 0.0001. Means ± s.e.m. **H** Upper panel: Schematic showing the viral strategy used to inhibit LS^Nts^ neurons projecting to TU through an optogenetic approach. Lower panel: Representative images showing the expression of eNpHR-EYFP in LS^Nts^ neurons projecting to the TU. Scale bar: 200 μm. **I** Optogenetic inhibition of TU-projecting LS^Nts^ neurons had no effect on regular food intake. EYFP control group, *n* = 5; eNpHR group, *n* = 6. Two-way ANOVA (F_(1,18)_ = 0.2096, *P* > 0.05). Means ± s.e.m. **J** Optogenetic inhibition of TU-projecting LS^Nts^ neurons significantly increased the intake of Ensure. EYFP control group, *n* = 5; eNpHR group, *n* = 6. Two-way ANOVA (*F*_(1,18)_ = 5.341, *P* < 0.05) followed by Sidak’s post hoc test. **P < 0.01. Means ± s.e.m. **K** Representative images showing the in situ hybridization results for the neurotensin receptor 1 (NtsR1) mRNA signal in the SUM. **L** Schematic showing the experimental design for the local infusion of the Nts peptide into the SUM. **M** Quantification of 2-h intake of standard chow after saline (gray bar, *n* = 8) or Nts (blue bar, *n* = 8) administration to the SUM. Wilcoxon signed-rank test. ***P < 0.001. **N** Quantification of 2-h intake of high-fat food after saline (gray bar, *n* = 8) or Nts (blue bar, *n* = 8) administration to the SUM. Wilcoxon signed-rank test. **P < 0.01. **O** Average Ca^2+^ activity of LS^Nts^→TU recorded by fiber photometry during free feeding of regular food (left panel) or Ensure (right panel). Upper panel: Population average from 5 mice. Lower panel: Ca^2+^ activity in individual mice. **P** Average Ca^2+^ activity of the LS^Nts^→SUM circuit recorded by fiber photometry during free feeding of regular food (left panel) or Ensure (right panel). Upper panel: Population average from 3 mice. Lower panel: Ca^2+^ activity in individual mice.

To further explore the circuit mechanisms of feeding regulation by LS^Nts^ neurons, we systematically mapped the projections from LS^Nts^ neurons using SynaptoTag AAV, which coexpresses the red fluorescent protein tdTomato and enhanced green fluorescent protein (EGFP) fused to the synaptic vesicle protein synaptophysin [19]. We injected AAV-FLEX-tdTomato-T2A-synaptophysin-EGFP into the LS of *Nts-ires-cre* mice (Fig. 7A). Neurons infected with SynaptoTag AAV were filled with tdTomato in their cytoplasm and axon fibers and localized green fluorescent synaptophysin to efferent synapses. With this tracing strategy, we found that LS^Nts^ neurons made major synaptic connections with several brain regions, including the lateral and medial preoptic area (POA), tuberal nucleus (TU), anterior hypothalamic nucleus(AHN), and supramammillary nucleus (SUM) (Fig. 7B, C). We expressed ChR2 in LS^Nts^ neurons and performed whole-cell recording from TU neurons in slices to verify functional synaptic connections. Brief blue light stimulation of LS^Nts^ axonal terminals in the TU evoked robust picrotoxin-sensitive IPSCs from ~50% of recorded TU neurons (Fig. S7C–E), confirming functional GABAergic synaptic connections between LS^Nts^ and TU neurons.

To reveal the anatomical organization of LS^Nts^ projections, we injected the retrograde tracers CTB555 and CTB647/488 into the TU and other LS^Nts^ downstream targets and examined the overlap between CTB-labeled LS^Nts^ neurons projecting to distinct targets (Fig. S9). Only 6.0% of TU-projecting LS^Nts^ neurons projected to the POA, 8.3% of TU-projecting LS^Nts^ neurons projected to the AHN and 11.3% of TU-projecting LS^Nts^ neurons projected to the SUM (Fig. S9B–E), suggesting little overlap between TU-projecting and POA-projecting, AHN-projecting, SUM-projecting LS^Nts^ neurons. As expected, coinjection of CTB555 and CTB647 into the TU resulted in a high percentage of colabeling (92.9% for CTB555 and 66.0% for CTB647) (Fig. S9F). Using a similar method, we observed little overlap between every other pair of LS^Nts^ downstream projections (Fig. S9B), supporting a one-to-one projection pattern of LS^Nts^ neurons.

We examined which LS^Nts^ projection is critical in the regulation of hedonic feeding by injecting retroAAV-FLEX-FlpO into one of the downstream projection targets and AAV-fDIO-hM3D into the LS of *Nts-ires-Cre* mice (Fig. 7D). This strategy resulted in the selective expression of hM3D in LS^Nts^ neurons that project to a specific downstream target, and the number of hM3D-expressing LS^Nts^ neurons was comparable between different pathways (Fig. S7A, B). The activation of LS^Nts^→POA, LS^Nts^→AHN and LS^Nts^→SUM circuits by CNO injection significantly suppressed food intake, regardless of the food type (Fig. 7E–G). However, activation of the LS^Nts^→TU pathway specifically suppressed the consumption of palatable high-fat and high-sucrose food but not standard chow (Fig. 7E–G). Activation of the LS^Nts^ →TU pathway had no effect on general locomotor activity and anxiety-like behavior in the open field test (Fig. S7H–J). Because of the specific role of the LS^Nts^→TU pathway in hedonic feeding, we then tested whether silencing this pathway enhanced hedonic feeding. We employed a pathway-specific optogenetic strategy to inhibit the LS^Nts^→TU pathway (Fig. 7H). Indeed, inhibition of the LS^Nts^→TU pathway promoted feeding with palatable Ensure but not standard chow (Fig. 7I, J). The somatostatin (SST)-positive neurons in the TU have been recently reported to mediate context-driven excessive feeding, mostly hedonic feeding [8]. We also examined the distribution of LS^Nts^ axonal terminals in the TU and detected extensive overlap between synaptophysin-positive synaptic boutons from LS^Nts^ and SST-positive neurons in the TU (Fig. S7F, G).

We examined whether downstream projection areas of LS^Nts^ neurons express neurotensin receptors and thus potentially mediate the anorectic effect of neurotensin signaling by performing in situ hybridization to detect the neurotensin receptor 1 (NtsR1) mRNA in the 4 major projection targets. We observed a robust NtsR1 mRNA signal in the SUM, while little or no NtsR1 mRNA was detected in POA, AHN and TU. (Figs. 7K and S8A). Local infusion of neurotensin peptide (1 μg) into the SUM suppressed feeding, irrespective of the food type, indicating that the neurotensin signal in SUM is sufficient to suppress overall feeding (Fig. 7L–N). In contrast, the infusion of neurotensin into the TU had no effect of feeding (Fig. S8A–C), consistent with the absence of neurotensin receptor expression. To examine how neurotensin affects neuronal activity, we performed whole-cell patch clamp recording from TU and SUM neurons. In slice prepration, application of neurotensin (2 μM) increased the action potential firing in 3/6 recorded neurons in SUM (Fig. S8H). However, no neurons in TU (0/7) showed response to neurotensin application in slice (Fig. S8G). We also infused neurotensin (1 μg) locally into SUM in vivo and observed robust c-fos expression (Fig. S8D–F), further confirming that neurotensin signal activate SUM neurons.

To examine the possibility that excitatory and inhibitory responses arise from LS^Nts^ neurons projecting to distinct downstream targets, we recorded Ca^2+^ dynamics from LS^Nts^→TU and LS^Nts^→SUM pathways by expressing GCaMP in LS^Nts^ neurons projecting TU or SUM. Similar to the LS^Nts^ population, LS^Nts^→TU exhibited biphasic responses during free Ensure feeding (Fig. 7O). However, LS^Nts^→SUM showed a pure inhibitory response, and the response amplitude was larger when mice were consuming Ensure than when they consumed regular food (Fig. 7P).

### Activation of LS^Nts^ neurons or the LS^Nts^→TU pathway prevents high-fat diet-induced obesity

**Fig. 8:**
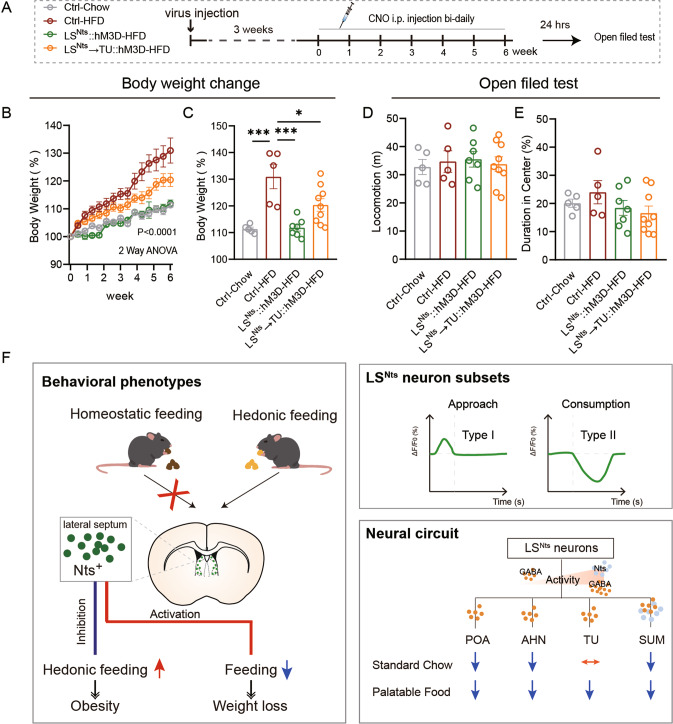
Activation of LS^Nts^ neurons prevents high-fat diet-induced obesity. **A** Schematic showing the experimental design for chronic chemogenetic activation of LS^Nts^ neurons in a high-fat diet-induced obesity model. **B** Changes in the body weight of control mice fed standard chow (gray, *n* = 5), control mice fed a high-fat diet (red, *n* = 5), LS^Nts^::hM3D mice fed a high-fat diet (green, *n* = 7) and LS^Nts^→TU::hM3D mice fed a high-fat diet (orange, *n* = 9) over several weeks. Two-way ANOVA (F_(3,22)_ = 13.74, *P* < 0.0001). Means ± s.e.m. **C** Changes in the body weight of control mice fed standard chow (gray, *n* = 5), control mice fed a high-fat diet (red, *n* = 5), LS^Nts^::hM3D mice fed a high-fat diet (green, *n* = 7) and LS^Nts^→TU::hM3D mice fed a high-fat diet (orange, *n* = 9) after 6 weeks. Two-way ANOVA (F_(3,22)_ = 11.4, *P* < 0.001) followed by Tukey’s post hoc test. *P < 0.05 and ***P < 0.001. Means ± s.e.m. **D** The average locomotor activity of control mice fed standard chow (gray, *n* = 5), control mice fed a high-fat diet (red, *n* = 5), LS^Nts^::hM3D mice fed a high-fat diet (green, *n* = 7) and LS^Nts^→TU::hM3D mice fed a high-fat diet (orange, *n* = 9). One-way ANOVA (F_(3,22)_ = 0.15, *P* > 0.05). Means ± s.e.m. **E** The duration in the center of the open field test for control mice fed standard chow (gray, *n* = 5), control mice fed a high-fat diet (red, *n* = 5), LS^Nts^::hM3D mice fed a high-fat diet (green, *n* = 7) and LS^Nts^→TU::hM3D mice fed a high-fat diet (orange, *n* = 9). One-way ANOVA (F_(3,22)_ = 1.19, *P* > 0.05). Means ± s.e.m. **F** Working model of the molecular and circuitry mechanism by which LS^Nts^ neurons regulate hedonic feeding and body weight.

Activation of LS^Nts^ neurons suppressed feeding, while the manipulation of LS^Nts^ neurons had no effect on energy expenditure. We wondered whether enhancing LS^Nts^ neuronal activity would prevent high-fat diet-induced obesity, and we chronically activated LS^Nts^ neurons with a chemogenetic approach (Fig. 8A). In control mice, body weight increased rapidly upon the introduction of the high-fat diet and increased by 37% ± 4% after 6 weeks (Fig. 8B, red line), while the body weight of mice maintained on a standard chow diet only increased by 14% ± 1% (Fig. 8B, gray line). Chemogenetic activation of LS^Nts^ neurons by a daily IP injection of CNO reversed the increase in body weight (10% ± 1%) (Fig. 8B, green line). Using an intersectional viral strategy, we selectively transduced hM3D in TU-projecting LS^Nts^ neurons by injecting retroAAV-FLEX-FlpO into the TU and AAV-fDIO-hM3D into the LS of *Nts-ires-Cre* mice. Chemogenetic activation of LS^Nts^→TU also significantly reduced the increase in body weight induced by a high-fat diet (21% ± 3%) (Fig. 8B, orange line). Chronic activation of the LS^Nts^→TU pathway had no effect on locomotor activity or anxiety-related behavior in an open-field test (Fig. 8D, E). Based on these results, activation of LS^Nts^ neurons or the LS^Nts^→TU circuit is sufficient to reduce high-fat diet-induced obesity.

## Discussion

In the present study, we identified a group of neurotensin-expressing GABAergic neurons in the LS that play a critical role in regulating hedonic feeding. Silencing LS^Nts^ neurons promoted hedonic feeding, which was mediated by GABAergic projections to the TU. Activation of LS^Nts^ neurons suppressed overall food consumption, which was mediated by projections to the SUM, AHN and POA. Neurotensin signaling in the SUM is sufficient to suppress overall feeding. The identification of the precise molecules, cell types and circuitry involved in regulating hedonic feeding might aid in the development of better antiobesity drugs, given the undesired side effects of current drugs targeting homeostatic circuits.

The LS has been implicated in various physiological processes, including stress and anxiety [16, 29–31]. The LS contains many molecularly distinct cell types [32]. An intriguing hypothesis is that distinct cell types contribute to different physiological functions. A subset of LS neurons that express Crhr2 (LS^Crhr2^) has been shown to mediate persistent stress-induced anxiety behavior [16]. Our results reveal a critical role for LS^Nts^ neurons in modulating hedonic feeding but not anxiety, further supporting the hypothesis that distinct neuronal subtypes in the LS mediate different physiological processes.

LS^Nts^ neurons are a subset of GABAergic neurons in the LS that express neurotensin. Our results showed that LS^Nts^ neurons release GABA to act on downstream brain targets, as brief optogenetic activation of LS^Nts^ neurons evoked robust picrotoxin-sensitive inhibitory postsynaptic currents. Chemogenetic activation of neurotensin-expressing neurons causes the release of neurotensin to downstream brain areas [33]. Thus, LS^Nts^ neurons release both the canonical neurotransmitter GABA and the peptide neurotensin. Using CRISPR/Cas9-mediated gene knockdown, we revealed distinct roles for GABA and neurotensin signaling in modulating hedonic feeding. At the basal physiological level, GABA signaling specifically suppressed hedonic feeding, as vGAT knockdown promoted the intake of palatable food but not regular chow. However, chemogenetic activation of LS^Nts^ still suppressed the intake of both palatable and chow food in vGAT knockdown mice, suggesting the involvement of neurotensin signaling in suppressing overall feeding. Using an in situ hybridization technique, we detected the expression of neurotensin receptor 1 in the SUM (Fig. 7K), which is one of the downstream projection targets of LS^Nts^ neurons. Furthermore, we showed that a local infusion of neurotensin into the SUM suppressed the intake of both palatable food and regular chow, suggesting the involvement of the neurotensin signal in the LS^Nts^→SUM pathway in suppressing overall feeding.

A recent study reported that neurotensin neurons in LS are linked to appetite suppression [31]. LS^Nts^ neurons were activated by stress and chemogenetic activation of LS^Nts^ led to general food suppression, thus this study suggested an important role for LS^Nts^ neurons in contributing to stress-induced food suppression [31]. However, the anorectic effect observed in our experiment is unlikely to be due to stress-induced food suppression for the reasons described below. First, in our hedonic feeding model, mice had been habituated to a palatable diet for at least 3 days; thus, the effect of stress was likely minimal. Second, TeNT-mediated synaptic silencing of LS^Nts^ increased body the weight of mice fed a high-fat diet but had no effect on the body weight of mice fed a regular chow diet. This difference was not attributed to stress, as both animals were raised under the same condition and had similar stress levels. Instead, this result indicates that LS^Nts^ neurons limit hedonic feeding under physiological conditions. Third, using in vivo miniature microscopic Ca^2+^ imaging, we observed that 31% of LS^Nts^ neurons were activated while 32% of neurons were inhibited during free feeding of palatable food. Moreover, we also examined the responses of the same group of LS^Nts^ neurons to stress (footshock) and found that the majority of LS^Nts^ neurons (74%) were activated by stress (Fig. S6I). Thus, strong activation of LS^Nts^ neurons by stress very likely promotes neurotensin release to suppress overall food intake. Although LS^Nts^ neurons may link stress to food suppression, our studies revealed a significant role for LS^Nts^ neurons in modulating hedonic feeding in a nonstressful environment. Furthermore, our miniature microscopic Ca^2+^ imaging experiments revealed the heterogeneity of LS^Nts^ neurons during feeding, which has not been characterized in the previous literature.

Our circuit tracing experiments revealed that LS^Nts^ neurons project to multiple downstream targets, including the POA, AHN, TU and SUM. While activating projections to the POA, AHN and SUM suppressed overall feeding, activation of the LS^Nts^→TU circuit specifically inhibited the consumption of palatable food. These results suggest a unique role for the LS^Nts^→TU pathway in regulating hedonic feeding. Interestingly, SST-positive neurons in the tuberal nucleus have been recently reported to mediate environmental context-driven nonhomeostatic feeding, mostly hedonic feeding [8]. Our SynaptoTag-mediated anterograde tracing revealed robust contacts between synaptic boutons from LS^Nts^ neurons and SST-positive neurons in the TU. Thus, a plausible hypothesis is that the effect of the LS^Nts^ →TU circuit on suppressing hedonic feeding is at least partially mediated by SST neurons in the TU.

Our miniscope imaging experiment revealed two populations of LS^Nts^ neurons that were activated and inhibited during feeding. This finding explains the biphasic response observed in our fiber photometry experiment. The inhibited LS^Nts^ subpopulation exhibited a slower response latency and a wider response window than the activated LS^Nts^ subpopulation. The differences in temporal kinetics of those two LS^Nts^ populations resulted in biphasic responses at the population level. Our analysis of neuronal dynamics and perturbation experiments indicate a dominant effect of the inhibited LS^Nts^ subpopulation on food intake. Our results also reveal the heterogeneity of LS^Nts^ neurons and emphasize the importance of monitoring individual neuronal activity during physiological processes. In the future, an important goal is to determine whether these activated and inhibited LS^Nts^ subpopulations have different molecular profiles and whether they differ in their synaptic inputs and projection patterns.

In summary, our results identify a novel LS circuit that plays an important role in regulating hedonic feeding and obesity. Projections from the LS^Nts^ neurons to the TU suppress hedonic feeding via GABA signaling, while projections from the LS^Nts^ neurons to the POA, AHN and SUM suppress overall feeding. The neurotensin signal in the LS^Nts^→SUM pathway is sufficient to suppress overall feeding (Fig. 8F). These findings broaden our understanding of neural circuits underlying hedonic feeding beyond the classical mesolimbic dopaminergic reward system and will aid in developing interventions for excessive feeding driven by food palatability and resulting obesity.

## Supplementary information

Supplementary material

## References

[1] Finucane MM, Stevens GA, Cowan MJ, Danaei G, Lin JK, Paciorek CJ (2011). National, regional, and global trends in body-mass index since 1980: systematic analysis of health examination surveys and epidemiological studies with 960 country-years and 9.1 million participants. Lancet.

[2] Saper CB, Chou TC, Elmquist JK (2002). The need to feed: homeostatic and hedonic control of eating. Neuron.

[3] Friedman JM, Halaas JL (1998). Leptin and the regulation of body weight in mammals. Nature.

[4] Sternson SM, Eiselt AK (2017). Three pillars for the neural control of appetite. Annu Rev Physiol.

[5] Berthoud HR, Munzberg H, Morrison CD (2017). Blaming the brain for obesity: integration of hedonic and homeostatic mechanisms. Gastroenterology.

[6] Rossi MA, Stuber GD (2018). Overlapping brain circuits for homeostatic and hedonic feeding. Cell Metab.

[7] Stern SA, Azevedo EP, Pomeranz LE, Doerig KR, Ivan VJ, Friedman JM (2021). Top-down control of conditioned overconsumption is mediated by insular cortex Nos1 neurons. Cell Metab.

[8] Mohammad H, Senol E, Graf M, Lee CY, Li Q, Liu Q (2021). A neural circuit for excessive feeding driven by environmental context in mice. Nat Neurosci.

[9] Gao Q, Horvath TL (2007). Neurobiology of feeding and energy expenditure. Annu Rev Neurosci.

[10] Meye FJ, Adan RA (2014). Feelings about food: the ventral tegmental area in food reward and emotional eating. Trends Pharmacol Sci.

[11] Lutter M, Nestler EJ (2009). Homeostatic and hedonic signals interact in the regulation of food intake. J Nutr.

[12] Zhou QY, Palmiter RD (1995). Dopamine-deficient mice are severely hypoactive, adipsic, and aphagic. Cell.

[13] Sternson SM, Atasoy D (2014). Agouti-related protein neuron circuits that regulate appetite. Neuroendocrinology.

[14] Denis RG, Joly-Amado A, Webber E, Langlet F, Schaeffer M, Padilla SL (2015). Palatability Can Drive Feeding Independent of AgRP Neurons. Cell Metab.

[15] Christoffel DJ, Walsh JJ, Heifets BD, Hoerbelt P, Neuner S, Sun G (2021). Input-specific modulation of murine nucleus accumbens differentially regulates hedonic feeding. Nat Commun.

[16] Anthony TE, Dee N, Bernard A, Lerchner W, Heintz N, Anderson DJ (2014). Control of stress-induced persistent anxiety by an extra-amygdala septohypothalamic circuit. Cell.

[17] Corwin RL, Avena NM, Boggiano MM (2011). Feeding and reward: perspectives from three rat models of binge eating. Physiol Behav.

[18] Leinninger GM, Opland DM, Jo YH, Faouzi M, Christensen L, Cappellucci LA (2011). Leptin action via neurotensin neurons controls orexin, the mesolimbic dopamine system and energy balance. Cell Metab.

[19] Xu W, Sudhof TC (2013). A neural circuit for memory specificity and generalization. Science.

[20] Armbruster BN, Li X, Pausch MH, Herlitze S, Roth BL (2007). Evolving the lock to fit the key to create a family of G protein-coupled receptors potently activated by an inert ligand. Proc Natl Acad Sci USA.

[21] Platt RJ, Chen S, Zhou Y, Yim MJ, Swiech L, Kempton HR (2014). CRISPR-Cas9 knockin mice for genome editing and cancer modeling. Cell.

[22] Rau AR, Hentges ST (2017). The Relevance of AgRP Neuron-Derived GABA Inputs to POMC Neurons Differs for Spontaneous and Evoked Release. J Neurosci.

[23] Cui G, Jun SB, Jin X, Pham MD, Vogel SS, Lovinger DM (2013). Concurrent activation of striatal direct and indirect pathways during action initiation. Nature.

[24] Mezuk B, Chen Y, Yu C, Guo Y, Bian Z, Collins R (2013). Depression, anxiety, and prevalent diabetes in the Chinese population: findings from the China Kadoorie Biobank of 0.5 million people. J Psychosom Res.

[25] Gong R, Xu S, Hermundstad A, Yu Y, Sternson SM (2020). Hindbrain double-negative feedback mediates palatability-guided food and water consumption. Cell.

[26] Boyden ES, Zhang F, Bamberg E, Nagel G, Deisseroth K (2005). Millisecond-timescale, genetically targeted optical control of neural activity. Nat Neurosci.

[27] Gradinaru V, Thompson KR, Deisseroth K (2008). eNpHR: a Natronomonas halorhodopsin enhanced for optogenetic applications. Brain Cell Biol.

[28] Ghosh KK, Burns LD, Cocker ED, Nimmerjahn A, Ziv Y, Gamal AE (2011). Miniaturized integration of a fluorescence microscope. Nat Methods.

[29] Wirtshafter HS, Wilson MA (2021). Lateral septum as a nexus for mood, motivation, and movement. Neurosci Biobehav Rev.

[30] Sheehan TP, Chambers RA, Russell DS (2004). Regulation of affect by the lateral septum: implications for neuropsychiatry. Brain Res Brain Res Rev.

[31] Azevedo EP, Tan B, Pomeranz LE, Ivan V, Fetcho R, Schneeberger M (2020). A limbic circuit selectively links active escape to food suppression. Elife.

[32] Risold PY, Swanson LW (1997). Chemoarchitecture of the rat lateral septal nucleus. Brain Res Brain Res Rev.

[33] Patterson CM, Wong JM, Leinninger GM, Allison MB, Mabrouk OS, Kasper CL (2015). Ventral tegmental area neurotensin signaling links the lateral hypothalamus to locomotor activity and striatal dopamine efflux in male mice. Endocrinology.

