# Supplementary material for: A circuit from lateral septum neurotensin neurons to tuberal nucleus controls hedonic feeding
Source: Mol Psychiatry. 2022 Aug 26;27(12):4843–60. doi: 10.1038/s41380-022-01742-0 (PMC9763109; doi:10.1038/s41380-022-01742-0)
Supplement: Supplementary file 1 — Supplementary material [file 41380_2022_1742_MOESM1_ESM.docx]

**A circuit from lateral septum neurotensin neurons to tuberal nucleus**

**controls hedonic feeding**

Zijun Chen^1,#^, Gaowei Chen^1,2,#^, Jiafeng Zhong^1,2^, Shaolei Jiang^3,4^, Shishi Lai^1^, Hua Xu^1^, Xiaofei Deng^1^, Fengling Li^1^, Shanshan Lu^1,2^, Kuikui Zhou^1,5^, Changlin Li^6^, Zhongdong Liu^3^, Xu Zhang^2,6,7^, Yingjie Zhu^1,2,5,8,9,10,*^

^1^ Shenzhen Key Laboratory of Drug Addiction, Shenzhen Neher Neural Plasticity Laboratory, the Brain Cognition and Brain Disease Institute, Shenzhen Institute of Advanced Technology, Chinese Academy of Sciences; Shenzhen-Hong Kong Institute of Brain Science-Shenzhen Fundamental Research Institutions, Shenzhen, 518055, China.

^2^ University of Chinese Academy of Sciences, Beijing, 100049, China.

^3^ Henan University of Technology, Henan, 450001, China.

^4^ University of Shanghai for Science and Technology, Shanghai, 200093, China.

^5^ Faculty of Life and Health Sciences, Shenzhen Institute of Advanced Technology, Chinese Academy of Sciences, Shenzhen, 518055, China.

^6^ Guangdong Institute of Intelligence Science and Technology, Hengqin District, Zhuhai, Guangdong, 519031, China.

^7^ Research Unit of Pain Medicine, Chinese Academy of Medical Sciences; SIMR Joint Lab of Drug Innovation, Shanghai Advanced Research Institute, Chinese Academy of Sciences, Shanghai 201210, China.

^8^ CAS Center for Excellence in Brain Science and Intelligence Technology, Chinese Academy of Sciences, Shanghai, 200031, China.

^9^ CAS Key Laboratory of Brain Connectome and Manipulation, the Brain Cognition and Brain Disease Institute (BCBDI), Shenzhen Institute of Advanced Technology (SIAT), Chinese Academy of Sciences, Shenzhen, 518055, China.

^10^ Lead Contact

^#^ These authors contributed equally to this work.

*Correspondence: [yj.zhu1@siat.ac.cn](mailto:yj.zhu1@siat.ac.cn) (Y.Z.)

**Supplemental Figures:**

**
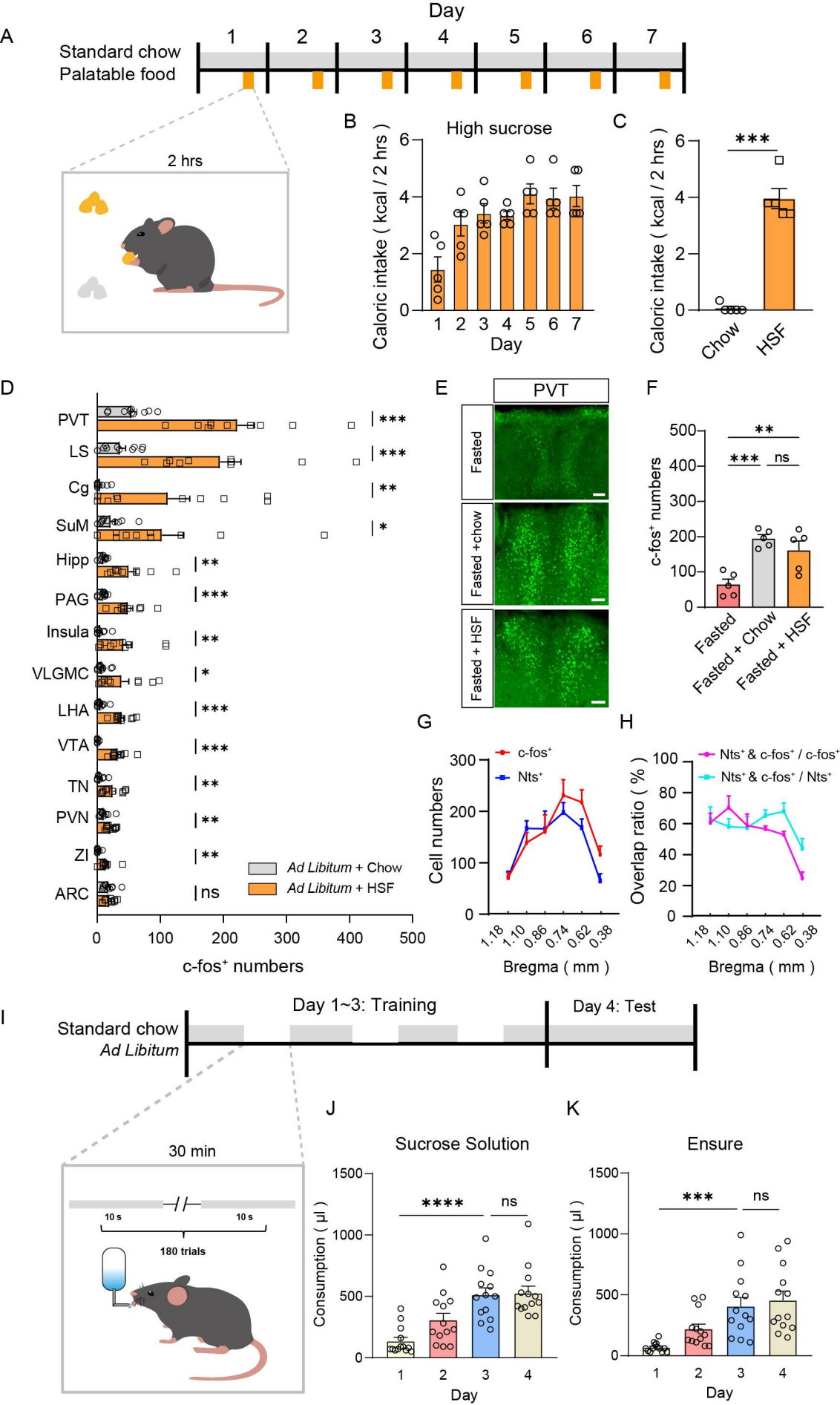
**

**Figure S1: Daily food intake and brain activations for mice under hedonic feeding protocol.**

1. Schematic showing protocol for hedonic feeding: mice had limited access to palatable food (2 hours per day), while *ad libitum* to standard chow for consecutive 7 days.
2. Total food intake during 2 hours per exposure to palatable food across day 1 to day 7. n = 5. Mean ± s.e.m.
3. Comparison of food intake for standard chow and palatable food during 2 hours exposure. n = 5. Wilcoxon signed-rank test. ***P < 0.001. Mean ± s.e.m.
4. Quantification of brain regions with significant difference in c-fos expression between control and hedonic feeding groups. Abbreviations are as follow: PVT, paraventricular nucleus of the thalamus; LS, lateral septum; Cg, cingulate cortex; SUM, supramammillary nucleus; PVN, paraventricular nucleus of hypothalamus; LHA, lateral hypothalamic area; ARC, arcuate nucleus; Insula, insula cortex; Hipp, hippocampus; PAG, the periaqueductal gray; VTA, ventral tegmental area; VLGMC, ventral lateral geniculate nucleus, magnocellular part; TU, tuberal nucleus; ZI, zona incerta. One-way ANOVA (P < 0.05) followed by post-hoc Tukey’s test. ns, no significant difference, *P < 0.05, **P < 0.01, ***P < 0.001. Mean ± s.e.m.
5. Representative images of c-fos immunostaining in PVT for fasted, fasted + chow and fasted + HSF group. Scale bar: 100 μm.
6. Number of c-fos^+^ neurons in PVT for fasted (n = 5), fasted + chow (n = 5) and fasted + HSF group (n = 5). One-way ANOVA (F_(2, 12)_ = 13.39, P < 0.05) followed by post-hoc Tukey’s test. ns, no significant difference, **P < 0.01, ***P < 0.001. Mean ± s.e.m.
7. Distribution of c-fos positive and neurotensin-positive neurons across anterior to posterior LS. (bregma 1.18 mm, n = 6; bregma 1.10 mm, n = 3; bregma 0.86 mm, n = 5; bregma 0.74 mm, n = 5; bregma 0.62 mm, n = 5; bregma 0.38 mm, n = 4).
8. Distribution of the portion of Nts^+^ neurons in c-fos^+^ neurons, and c-fos^+^ neurons in Nts^+^ neurons across anterior to posterior LS. (bregma 1.18 mm, n = 6; bregma 1.10 mm, n = 3; bregma 0.86 mm, n = 5; bregma 0.74 mm, n = 5; bregma 0.62 mm, n = 5; bregma 0.38 mm, n = 4).
9. Schematic showing protocol for hedonic feeding of liquid food.
10. Daily consumption of sucrose solution during experimental sessions. n = 13. One-way ANOVA (F_(3,48)_ = 13.08, P < 0.0001) followed by post-hoc Tukey’s test. n.s., no significant difference, ****P < 0.0001. Mean ± s.e.m.
11. Daily consumption of Ensure during experimental sessions. n = 13. One-way ANOVA (F_(3,48)_ = 10.07, P < 0.0001). n.s., no significant difference, ***P < 0.001. Mean ± s.e.m.


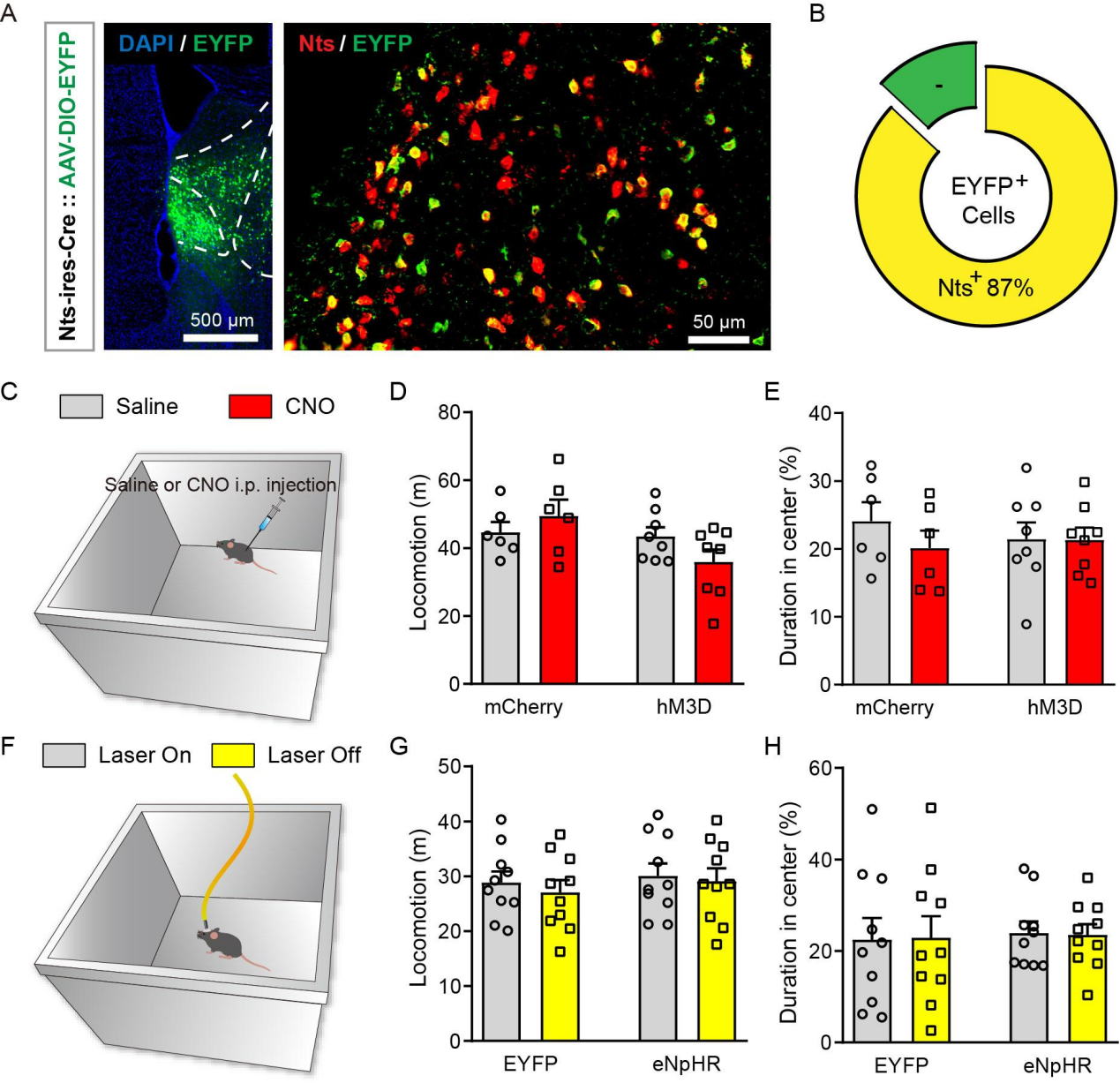


**Figure S2: Manipulation of LS^Nts^ neurons has no effect on locomotion and anxiety level**

1. Expression of EYFP in LS^Nts^ neurons. Left: Representative image of injection site and viral expression in the LS of Nts-ires-Cre mice. Scale bar: 500 μm. Right: Representative image showing the overlap of Nts (red) and EYFP (green) signal. Scale bar: 50 μm.
2. Quantification of the percentage of EYFP-positive cells that are Nts-positive.
3. Schematic showing chemogenetic activation of LS^Nts^ neurons at open field test.
4. CNO injection (2 mg/kg) had no effect on locomotion in both hM3D-expressing (n = 8) and mCherry-expressing (n = 6) mice. Two-way ANOVA (F_(1,24)_ = 3.02, P > 0.05). Mean ± s.e.m.
5. CNO injection (2 mg/kg) had no effect on the time spent in center in both hM3D-expressing (n = 8) and mCherry-expressing (n = 6) mice. Two-way ANOVA (F_(1,24)_ = 0.64, P > 0.05). Mean ± s.e.m.
6. Schematic showing optogenetic inhibition of LS^Nts^ neurons at open field test.
7. Yellow light stimulation had no effect on locomotion in both eNpHR-expressing (n = 10) and EYFP-expressing (n = 10) mice. Two-way ANOVA (F_(1,36)_ = 0.036, P > 0.05). Mean ± s.e.m.
8. Yellow light stimulation had no effect on the time spent in center in both eNpHR-expressing (n = 10) and EYFP-expressing (n = 10) mice. Two-way ANOVA (F_(1,36)_ = 0.013, P > 0.05). Mean ± s.e.m.


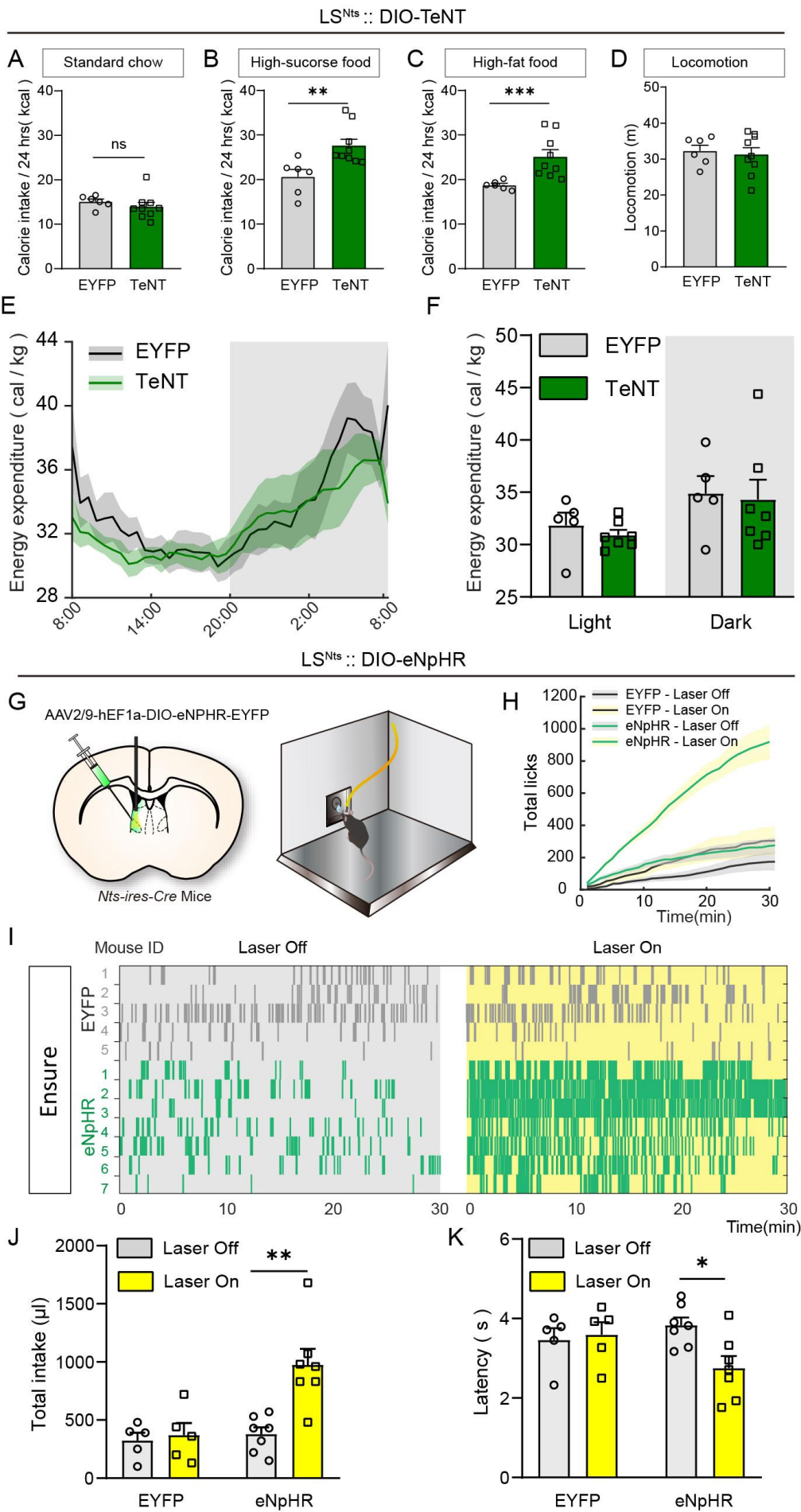


**Figure S3: Silencing of LS^Nts^ neurons promotes hedonic feeding with palatable food**

1. Quantification of 24 hours standard chow intake in EYFP- (gray bar, n = 6) and TeNT- (green bar, n = 9) expressing mice. Mann-Whitney U test. ns, no significant difference. Mean ± s.e.m.
2. Quantification of 24 hours high-sucrose food intake in EYFP- (gray bar, n = 6) and TeNT- (green bar, n = 9) expressing mice. Mann-Whitney U test. **P < 0.01. Mean ± s.e.m.
3. Quantification of 24 hours high-fat food intake in EYFP- (gray bar, n = 6) and TeNT- (green bar, n = 9) expressing mice. Mann-Whitney U test. ***P < 0.001. Mean ± s.e.m.
4. Quantification of locomotion in open field test for EYFP- (gray bar, n = 6) and TeNT- (green bar, n = 9) expressing mice.
5. Energy expenditure of EYFP- (gray line, n = 5) and TeNT- (green line, n = 7) expressing mice during 24 hrs.
6. Quantification of energy expenditure of EYFP- (gray bars, n = 5) and TeNT- (green bars, n = 7) expressing mice during light and dark phase. Two-way ANOVA(F_(1,20)_ = 0.014, P > 0.05).
7. Schematic showing optogenetic inhibition of LS^Nts^ neurons during feeding of liquid food.
8. Cumulative number of licks from an example animal showing that light stimulation promotes total licks during feeding of Ensure in eNpHR-expressing but not EYFP-expressing mice.
9. Lick pattern of EYFP-expressing (n = 5) and eNpHR-expressing (n = 7) mice during laser on and laser off period when consuming Ensure. Each vertical tick indicates a lick.
10. Light stimulation increased total intake of Ensure in eNpHR-expressing (n = 7) but not EYFP-expressing (n = 5) mice. Two-way ANOVA(F_(1,20)_ = 7.127, P < 0.05) followed by post-hoc Sidak’s test. ***P < 0.001. Mean ± s.e.m.
11. Light stimulation decreased latency to consume Ensure in eNpHR-expressing (n = 7) but not EYFP-expressing (n = 5) mice. Two-way ANOVA(F_(1,20)_ = 4.587, P < 0.05) followed by post-hoc Sidak’s test. *P < 0.05. Mean ± s.e.m.


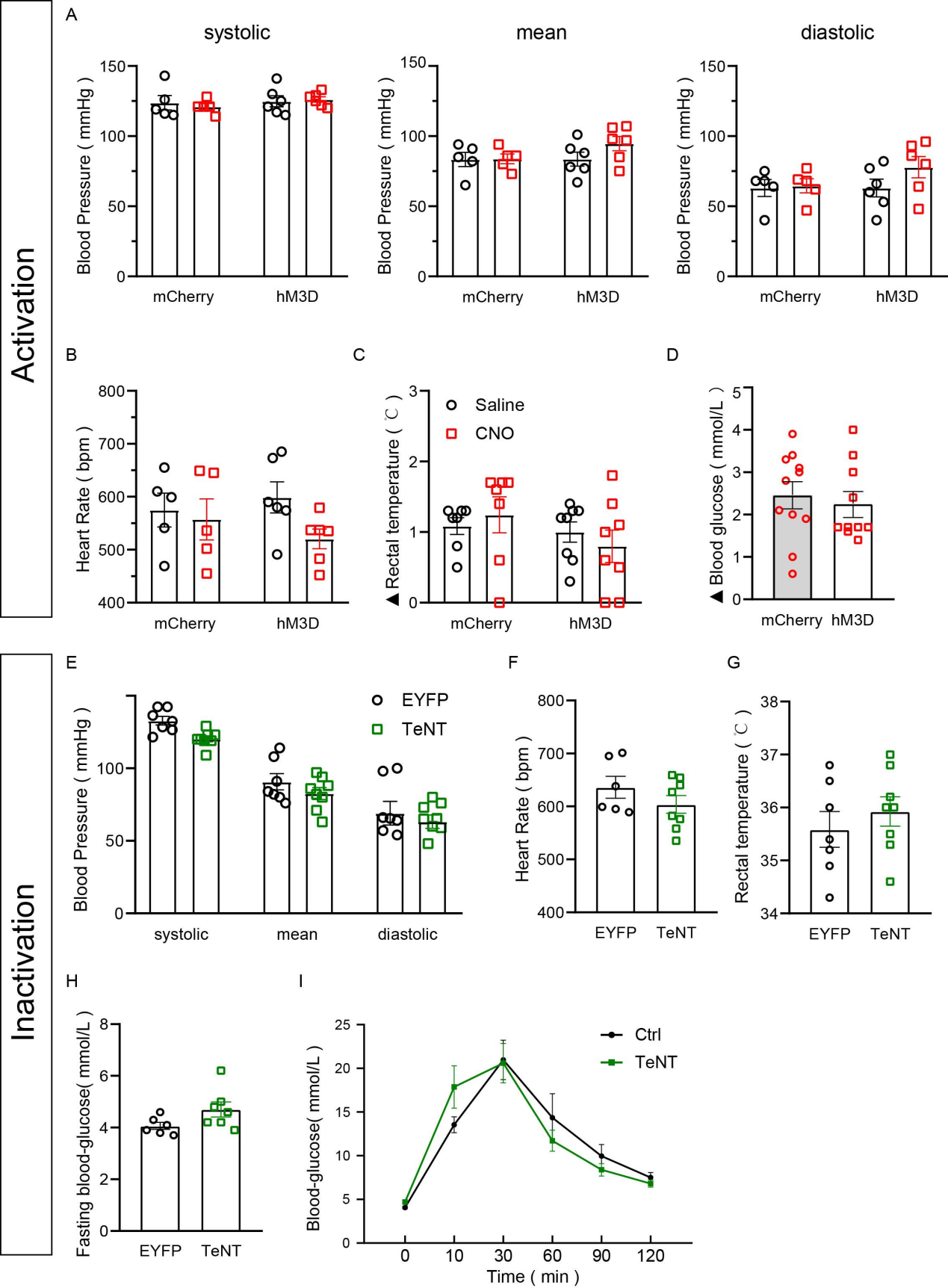


**Figure S4: Impacts of LS^Nts^ manipulation on physiological parameters.**

1. Effect of CNO injection (2 mg/kg) on systolic blood pressure (left), diastolic blood pressure (middle) and mean blood pressure (right) in hM3D-expressing (n = 6) and mCherry-expressing (n = 5) mice. Two-way ANOVA (systolic, F_(1,18)_ = 0.35, P > 0.05; mean, F_(1,18)_ = 1.21, P > 0.05; diastolic, F_(1,18)_ = 1.04, P > 0.05). Mean ± s.e.m.
2. Effect of CNO injection (2 mg/kg) on heart rate in hM3D-expressing (n = 6) and mCherry-expressing (n = 5) mice. Two-way ANOVA (F_(1,18)_ = 1.05, P > 0.05). Mean ± s.e.m.
3. Effect of CNO injection (2 mg/kg) on the rectal temperature in hM3D-expressing (n = 8) and mCherry-expressing (n = 7) mice. Two-way ANOVA (F_(1,26)_ = 0.84, P > 0.05). Mean ± s.e.m.
4. Effect of CNO injection on the blood glucose in hM3D-expressing (n = 10) and mCherry-expressing (n = 11) mice. Mann-Whitney U test. P > 0.05. Mean ± s.e.m.
5. The systolic blood pressure (left), mean blood pressure (middle) and diastolic blood pressure (right) in TeNT-expressing (n = 8) and EYFP-expressing (n = 7) mice. Two-way ANOVA (F_(2,39)_ = 0.31, P > 0.05). Mean ± s.e.m.
6. The heart rate for TeNT-expresing (n = 8) and EYFP-expressing (n = 6) mice. Mann-Whitney U test. P > 0.05. Mean ± s.e.m.
7. The rectal temperature for TeNT-expresing (n = 8) and EYFP-expressing (n = 7) mice. Mann-Whitney U test. P > 0.05. Mean ± s.e.m.
8. The fasted blood glucose for TeNT-expresing (n = 7) and EYFP-expressing (n = 6) mice. Mann-Whitney U test. P > 0.05. Mean ± s.e.m.
9. Oral glucose tolerance test for TeNT-expressing (n = 7) and EYFP-expressing (n = 6) mice. Two-way ANOVA (F_(1,11)_ = 0.0001, P > 0.05). Mean ± s.e.m.


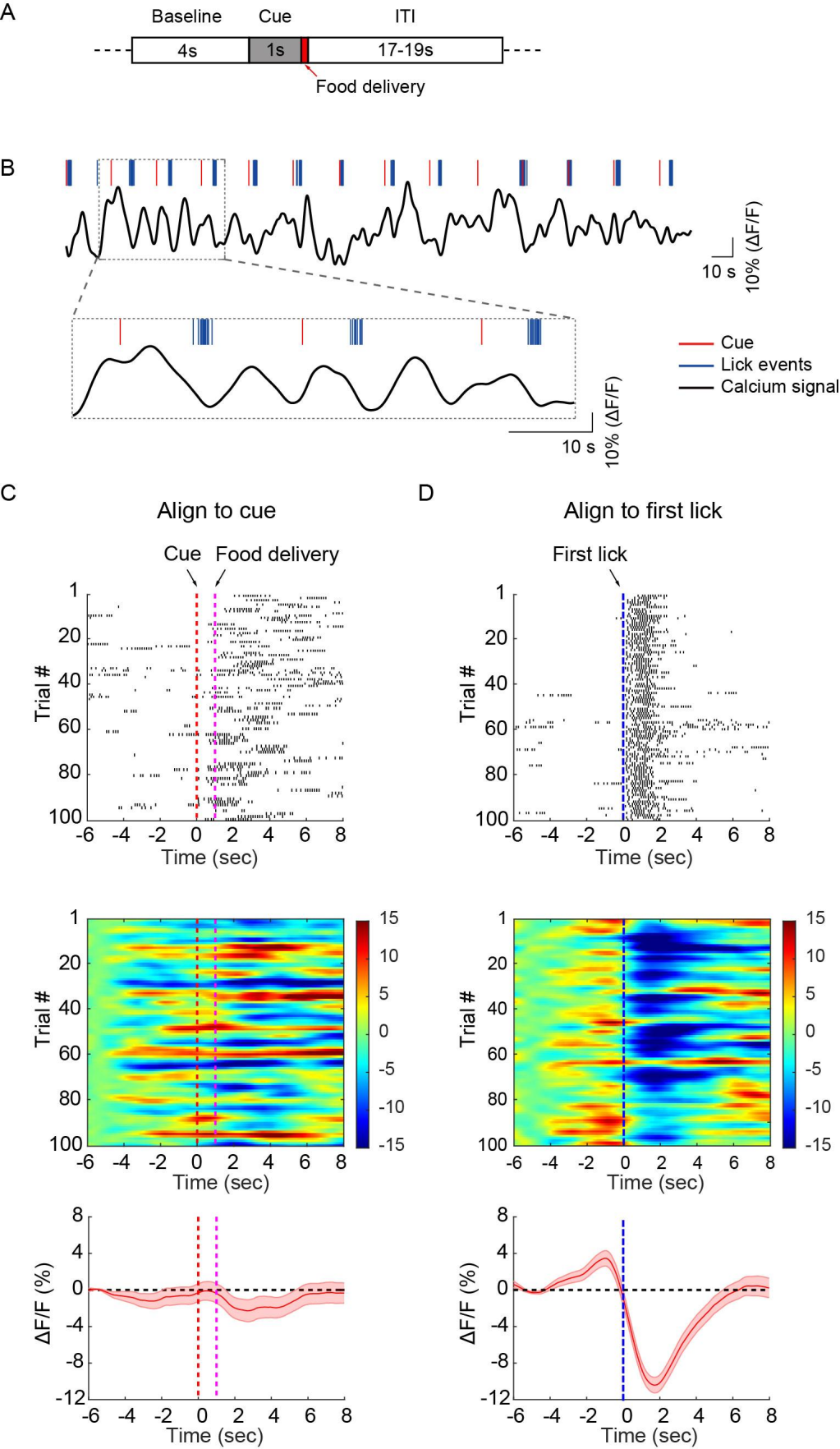


**Figure S5: Fiber photometry recording of LS^Nts^ activity during cue-conditioned feeding of Ensure**

1. Schematic showing protocol for cue-conditioned feeding protocol.
2. Representative trace of Ca^2+^ signal of LS^Nts^ during cue-conditioned feeding of Ensure. Red vertical bar indicate presentation of food-predicting cue. Blue vertical bar indicate licks.
3. The responses aligned to the onset of cue. Up: licking behavior aligned to cue of a representative mouse. Middle: heat map of population Ca^2+^ signal aligned to cue. Bottom: average Ca^2+^ response across trials aligned to cue.
4. The responses aligned to the first lick after the cue. Up: licking behavior aligned to first lick from a representative mouse. Middle: heat map of population Ca^2+^ signal aligned to first lick. Bottom: average Ca^2+^ response across trials aligned to first lick.


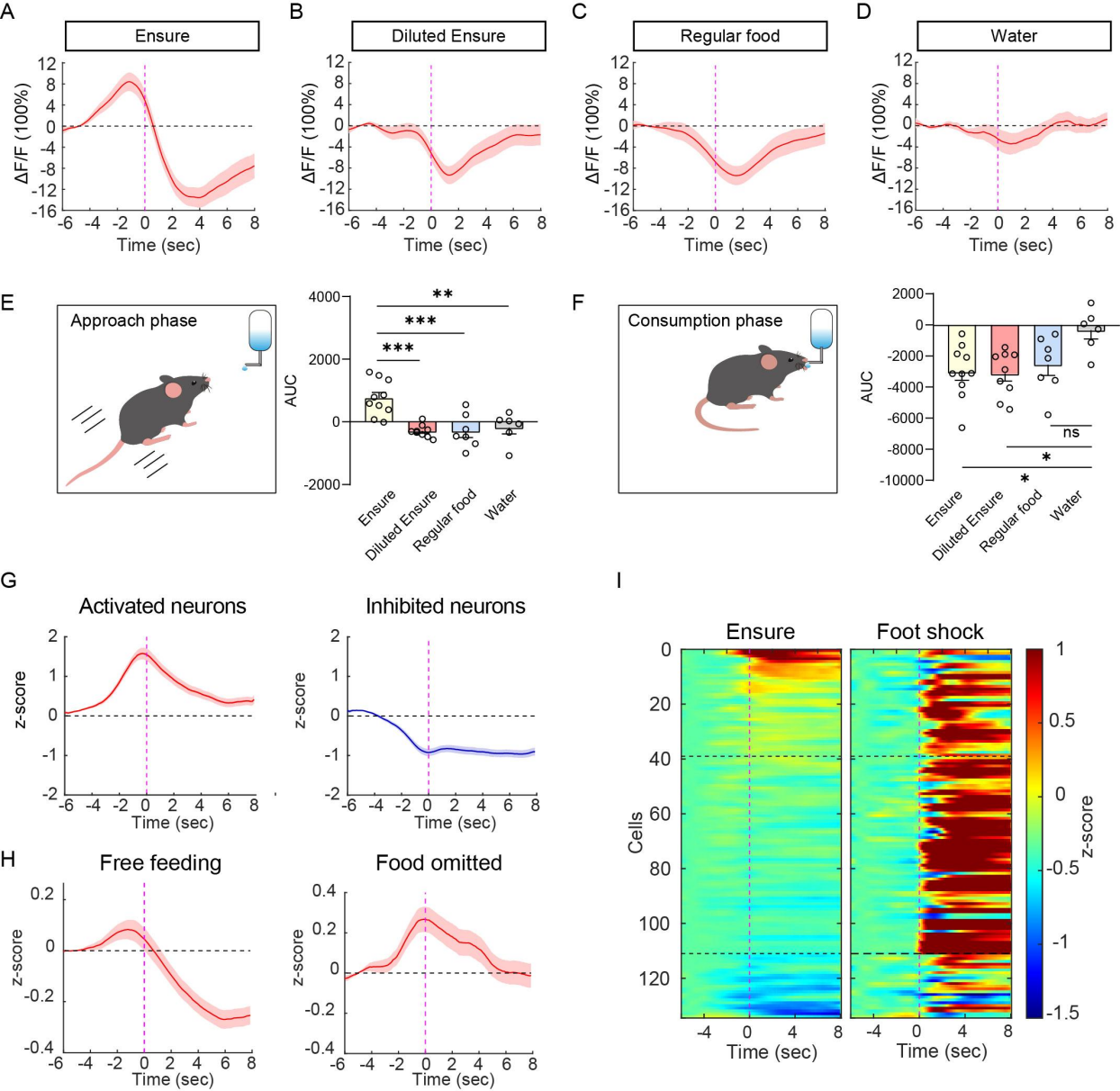


**Figure S6: Fiber photometry recording of LS^Nts^ activity during feeding of foods with different palatability; and miniature microscopy imaging of Ca^2+^ activity form LS^Nts^ neurons.**

1. Population Ca^2+^ activity of LS^Nts^ neurons recorded by fiber photometry during free feeding of Ensure. Vertical dashed line: first lick.
2. Population Ca^2+^ activity of LS^Nts^ neurons recorded by fiber photometry during free feeding of diluted Ensure. Vertical dashed line: first lick.
3. Population Ca^2+^ activity of LS^Nts^ neurons recorded by fiber photometry during free feeding of regular food. Vertical dashed line: first lick.
4. Population Ca^2+^ activity of LS^Nts^ neurons recorded by fiber photometry during consumption of water. Vertical dashed line: first lick.
5. Area under curve of Ca^2+^ activity during food approach phase. (n = 10/9/7/6). One-way ANOVA (F_(3, 28)_ = 11.3, P < 0.01) followed by post-hoc Tukey’s test. **P < 0.01, ***P < 0.001. Mean ± s.e.m.
6. Area under curve of Ca^2+^ activity during food consumption phase. (n = 10/9/7/6). One-way ANOVA (F_(3, 28)_ = 4.4, P < 0.01) followed by post-hoc Tukey’s test. ns, no significant difference, *P < 0.05. Mean ± s.e.m.
7. Average Ca^2+^ activity of activated (left, n = 88) and inhibited (right, n = 86) LS^Nts^ subpopulations in miniature microscopy Ca^2+^ imaging experiments.
8. Summated Ca^2+^ activity calculated from activated and inhibited LS^Nt^s subpopulations in free feeding of Ensure (left) and food omitted (right) condition in miniscope imaging experiments.
9. Ca^2+^ response of LS^Nts^ neurons to consumption of Ensure and footshock (n = 143 neurons from 3 mice) grouped by k-means clustering. Vertical dashed line: first lick or delivery of shook.


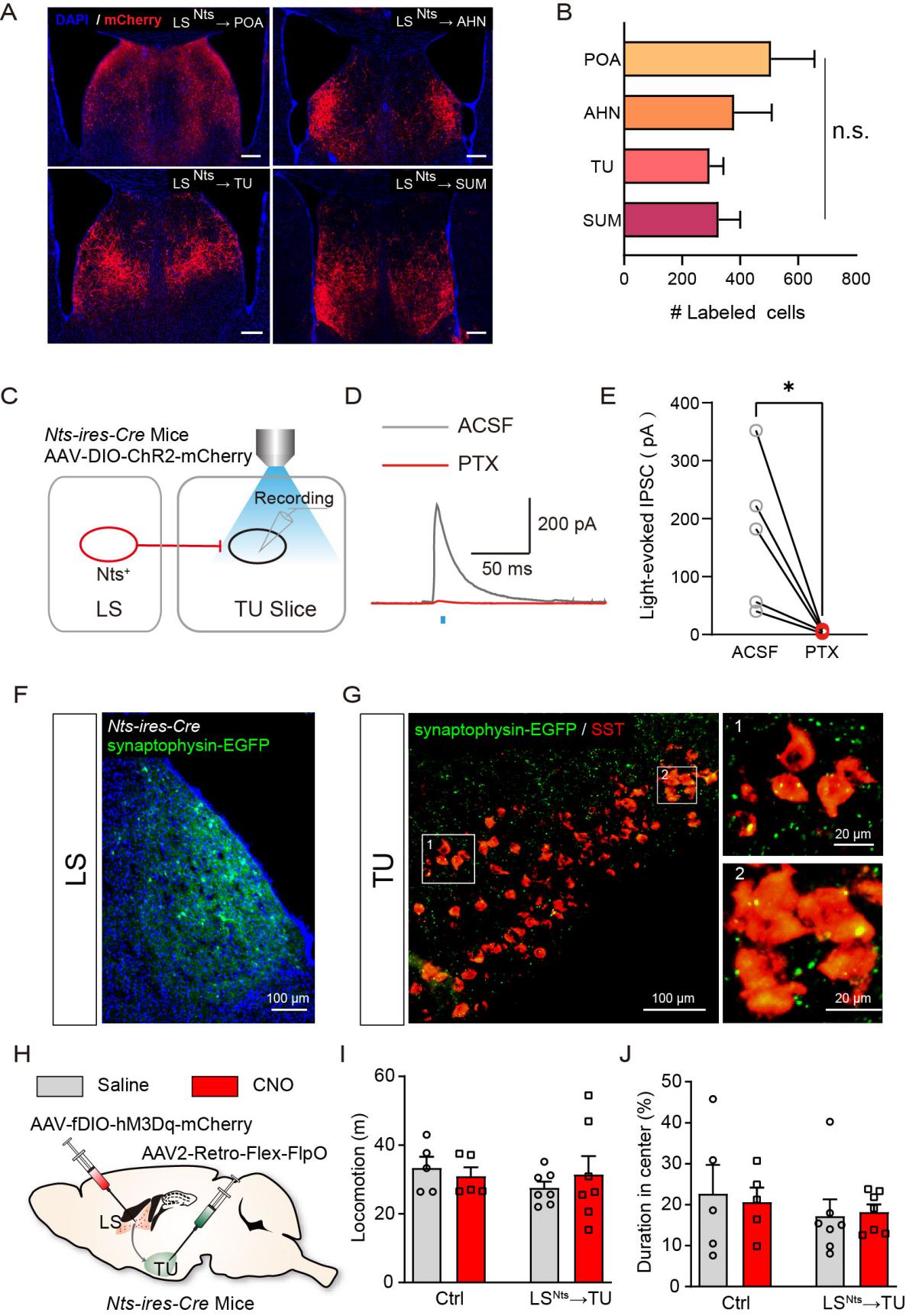


**Figure S7: Functional connectivity between LS^Nts^ and TU.**

1. Representative images showing the POA-projecting, AHN-projecting, TU-projecting and SUM-projecting LS^Nts^ neurons expressed hM3D-mCherry through intersectional viral strategy. Scale bar: 200 μm.
2. Quantification of number of labeled LS^Nts^ neurons that projecting to each downstream target. One-way ANOVA (F_(3, 13)_ = 0.70, P > 0.05). Mean ± s.e.m.
3. Schematic showing experiment design to record LS^Nts^🡪TU synaptic currents. Opsin ChR2 was expressed in LS^Nts^ neurons, while patch clamp recording was made from TU neurons in slice.
4. Blue light stimulation evoked robust inhibitory postsynaptic current (gray trace), which was blocked by picrotoxin (red trace).
5. Quantification of the amplitudes of light-evoked IPSCs with and without picrotoxin (n = 5). Wilcoxon signed-rank test. * P < 0.05.
6. Representative image showing the injection site in LS for SynaptoTag-mediated anterograde tracing.
7. Representative image showing the contacts between synaptophysin-positive synaptic boutons from LS^Nts^ neurons and SST-positive neurons in TU.
8. Schematic showing the viral strategy used to activate LS^Nts^ neurons projecting to TU through a chemogenetic approach.
9. CNO injection (2 mg/kg) had no effect on locomotion in both hM3D-expressing LS^Nts^🡪TU (red bar, n = 7) and mCherry-expressing control (gray bar, n = 5) mice. Two-way ANOVA (F_(1,10)_ = 1.51, P > 0.05). Mean ± s.e.m.
10. CNO injection (2 mg/kg) had no effect on duration in center in both hM3D-expressing LS^Nts^🡪TU (red bar, n = 7) and mCherry-expressing control (gray bar, n = 5) mice. Two-way ANOVA (F_(1,10)_ = 0.10, P > 0.05). Mean ± s.e.m.


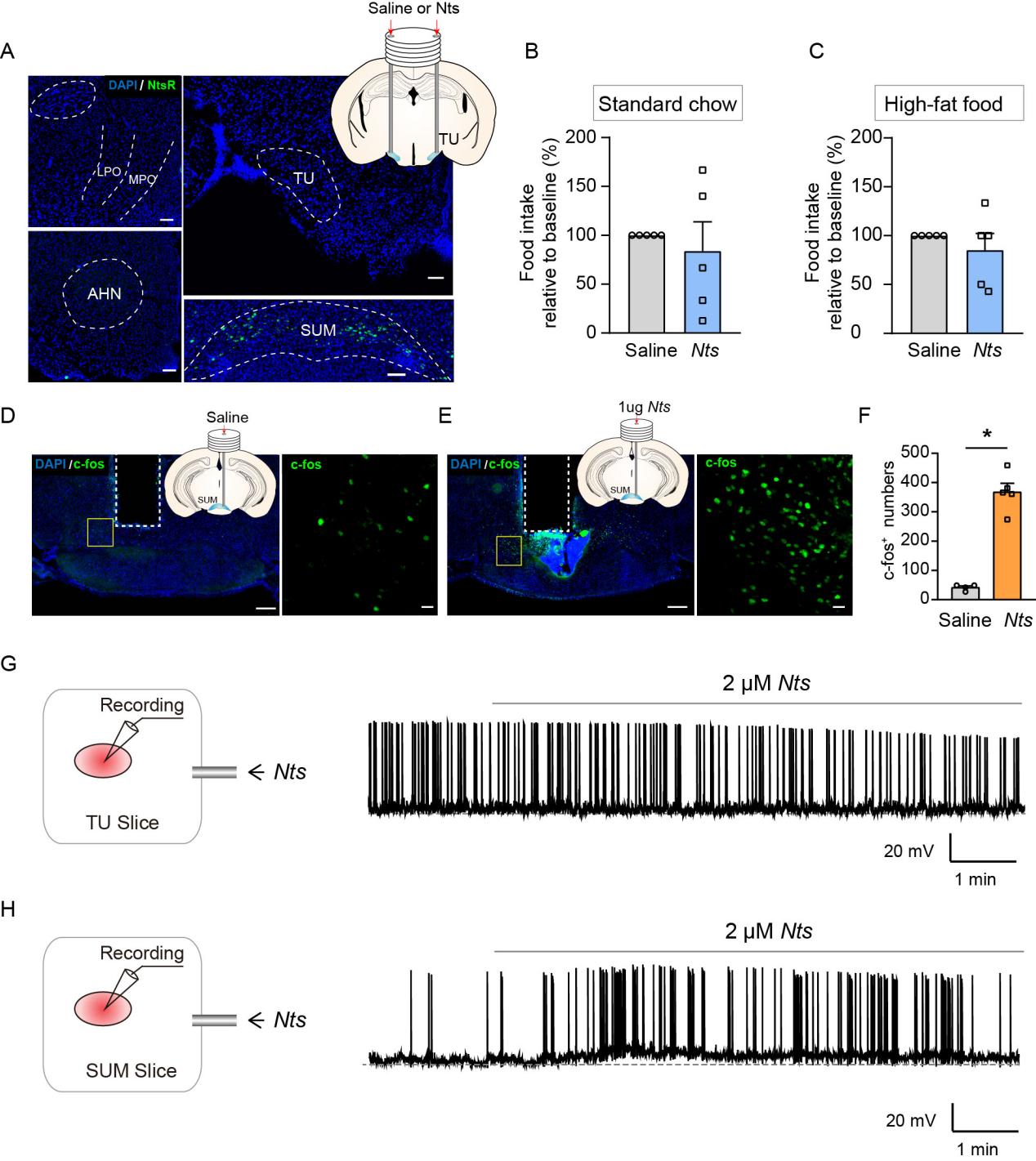


**Figure S8: The effect of neurotensin on food intake and neuronal activity**

1. Representative images showing the *in situ* hybridization results for detecting mRNA signal of neurotensin receptor 1 (NtsR1) in POA, TU, AHN and SUM. Inset: Schematic showing experimental design for local infusion of neurotensin peptide to TU.
2. Quantification of 2 hours intake of standard chow after saline (gray bar, n = 5) or neurotensin (blue bar, n = 5) administration to TU. Wilcoxon signed-rank test, P > 0.5.
3. Quantification of 2 hours intake of high-fat food after saline (gray bar, n = 5) or neurotensin (blue bar, n = 5) administration to TU. Wilcoxon signed-rank test, P > 0.5.
4. Left panel: Representative image showing the c-fos immunostaining after local infusion of saline to SUM. Inset: Schematic showing experimental design for local infusion of saline to SUM. Right panel: enlarged image for the area of yellow square in left panel.
5. Left panel: Representative image showing the c-fos immunostaining after local infusion of neurotensin to SUM. Inset: Schematic showing experimental design for local infusion of neurotensin to SUM. Right panel: enlarged image for the area of yellow square in left panel.
6. Quantification of number of c-fos^+^ neurons in SUM after local infusion of saline (gray bar, n = 4) or neurotensin (orange bar, n = 5). Mann-Whitney test, *P < 0.05.
7. Representive trace showing that the application of neurotensin in slice had no effect on spontaneous firing rate of TU neuron.
8. Representive trace showing that application of neurotensin in slice increased the action potential firing of SUM neuron.


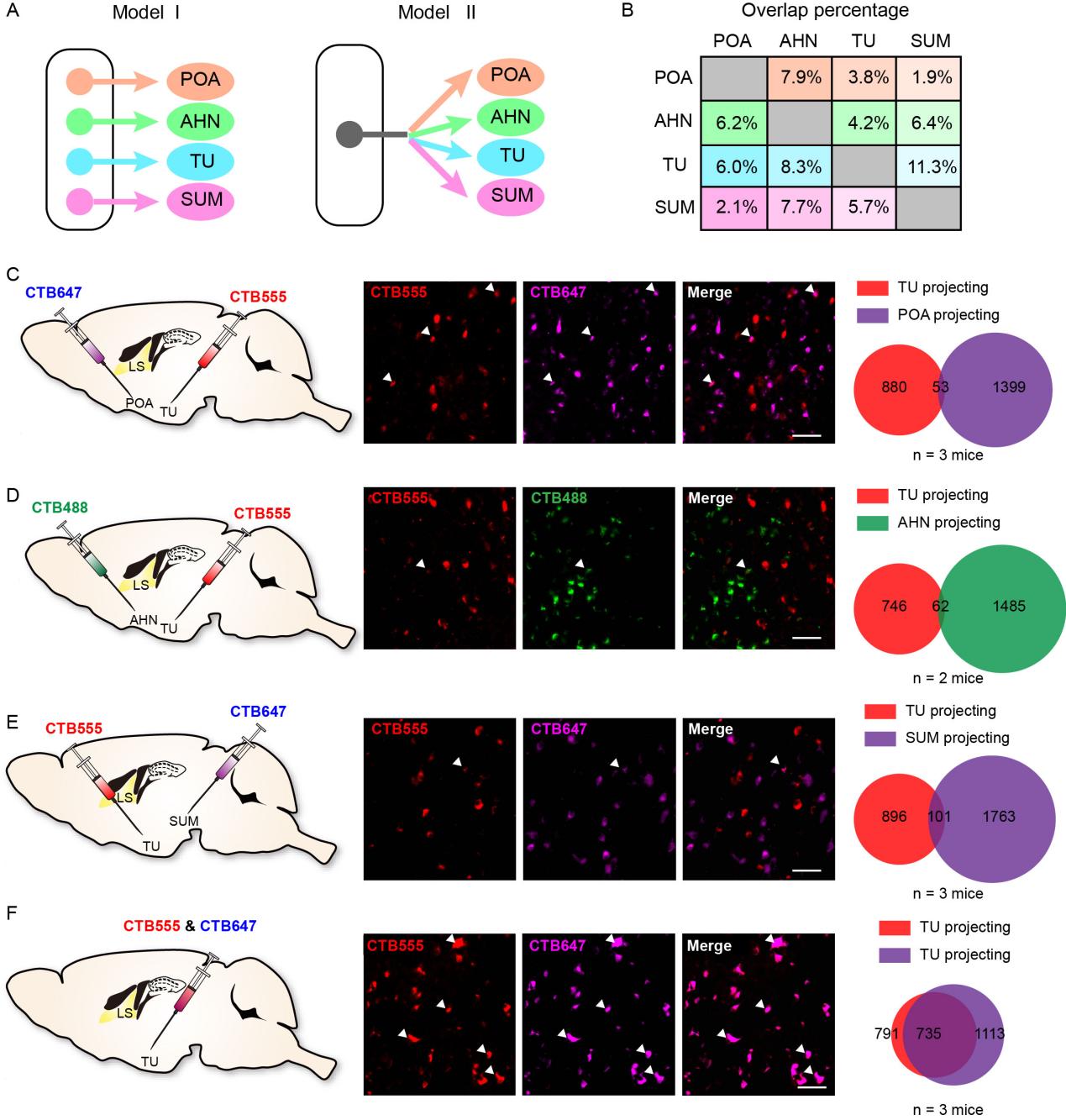


**Figure S9: One-to-one projection pattern of LS^Nts^ neurons**

1. Potential configurations for LS^Nts^ neurons axon projections. Model I: each LS^Nts^ neuron project to single downstream target; Model II: each LS^Nts^ neuron project to multiple downstream targets.
2. Overlap percentage of LS^Nts^ neurons projecting to different downstream targets.

C-E. Left, diagrams illustrating the injection of retrograde tracers in different downstream projection regions of LS^Nts^ neurons. Center, red LS neurons took up CTB555 injected in TU. Magenta or green LS neurons took up CTB647 or CTB488 injected in different projection sites. White arrows indicate the LS neurons positive for both CTB555 and CTB647 or CTB488. Scale bar, 100 μm. Right, Venn diagrams illustrate the quantifications from 8 mice.

F. Left, diagrams illustrating the injection of two retrograde tracers to TU. Center, red and magenta LS neurons took up CTB555 and CTB647 injected in TU. Scale bar, 50 μm. Right, Venn diagrams illustrate the quantifications from 3 mice.
